# Supplementary material for: A generalized framework for estimating snakebite underreporting using statistical models: A study in Colombia
Source: PLoS Negl Trop Dis. 2023 Feb 6;17(2):e0011117. doi: 10.1371/journal.pntd.0011117 (PMC9934346; doi:10.1371/journal.pntd.0011117)
Supplement: S1 Fig — (DOCX) [file pntd.0011117.s001.docx]

**
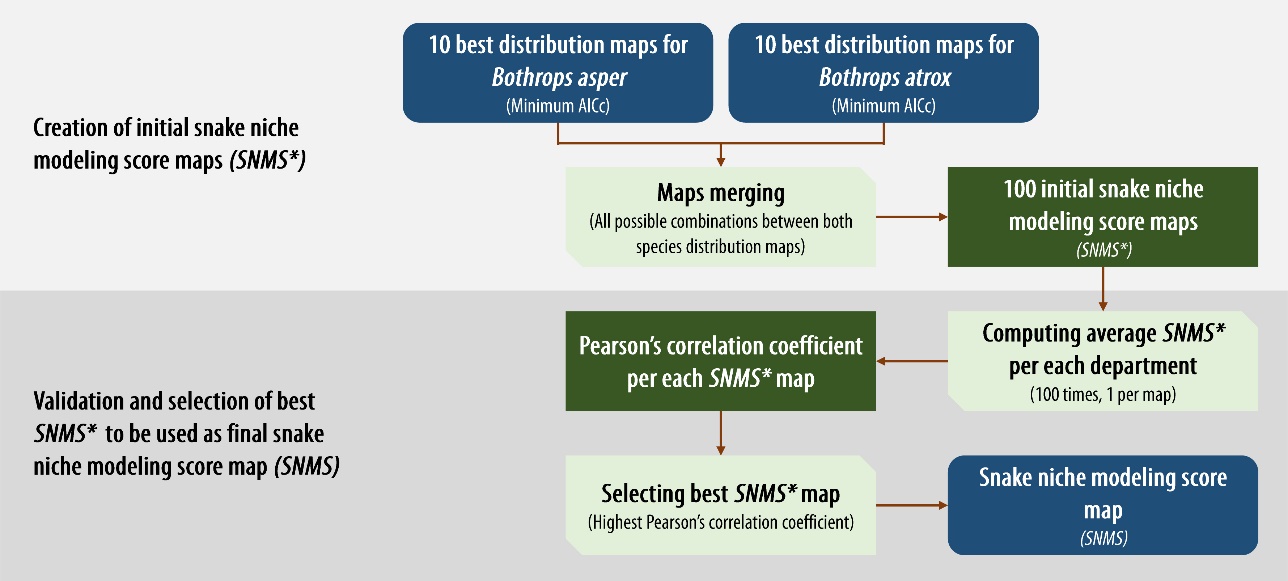
**

*Fig S1. Standard operating procedure for selecting the final snake niche modeling score map (SNMS) from the 100 initial snake niche modeling score maps (SNMS*)*
