# Supplementary figures and images for: A generalized framework for estimating snakebite underreporting using statistical models: A study in Colombia
Source: PLoS Negl Trop Dis. 2023 Feb 6;17(2):e0011117. doi: 10.1371/journal.pntd.0011117 (PMC9934346; doi:10.1371/journal.pntd.0011117)

**
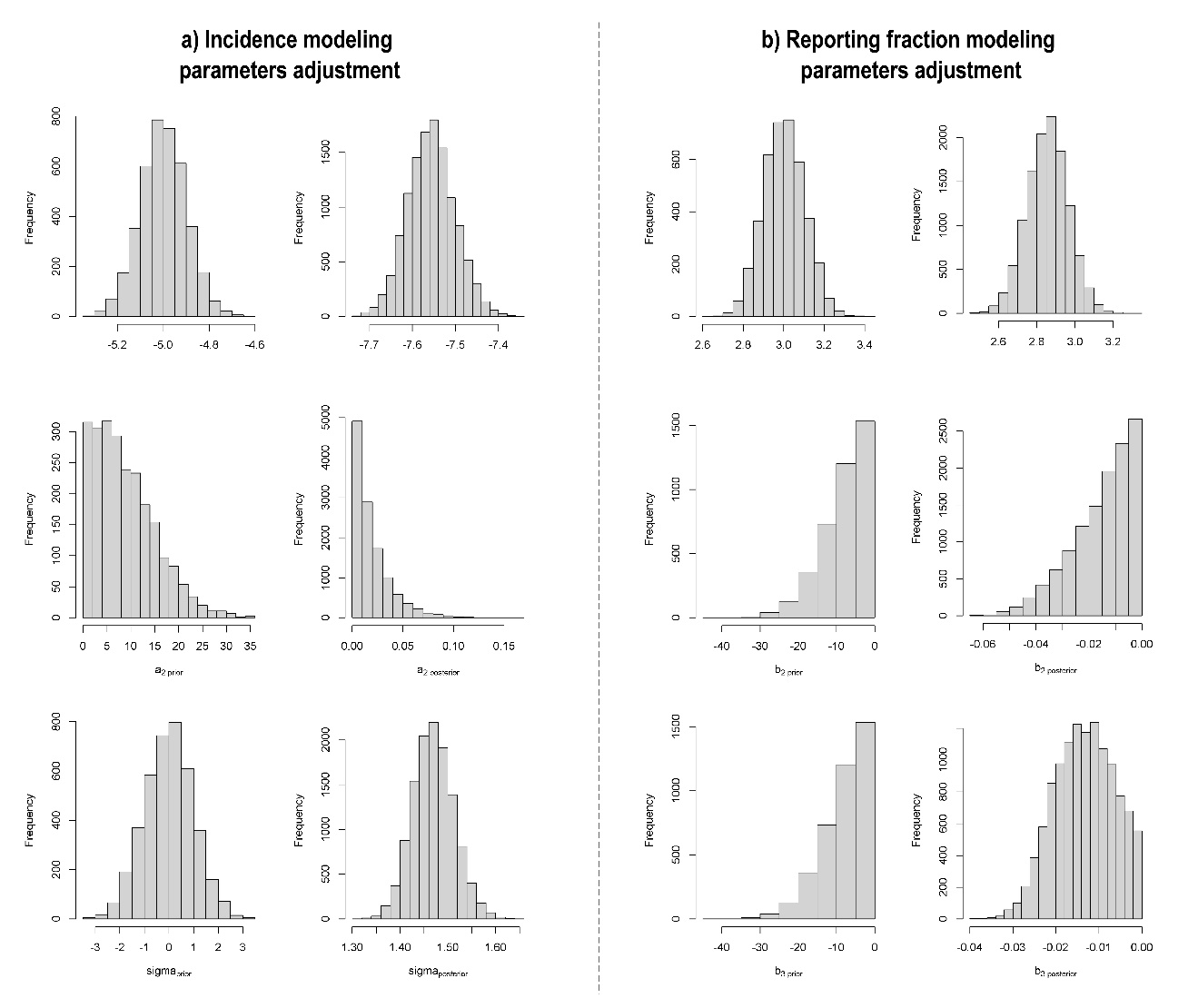
**

***Fig S2.*** *The prior and posterior distribution for model parameters after fitting convergence.*

Supplement: S2 Fig — (DOCX) [file pntd.0011117.s002.docx]
