# Supplementary material for: A generalized framework for estimating snakebite underreporting using statistical models: A study in Colombia
Source: PLoS Negl Trop Dis. 2023 Feb 6;17(2):e0011117. doi: 10.1371/journal.pntd.0011117 (PMC9934346; doi:10.1371/journal.pntd.0011117)
Supplement: S3 Fig — As we stated in the model, there is a positive correlation between both variables and underreporting, where low public health coverage and high poverty will increase underreporting fraction. Therefore, our estimation of underreporting varies between 5.68% and 37.83%. Credible intervals were obtained by computing the highest density intervals for posterior distribution. (DOCX) [file pntd.0011117.s003.docx]

**
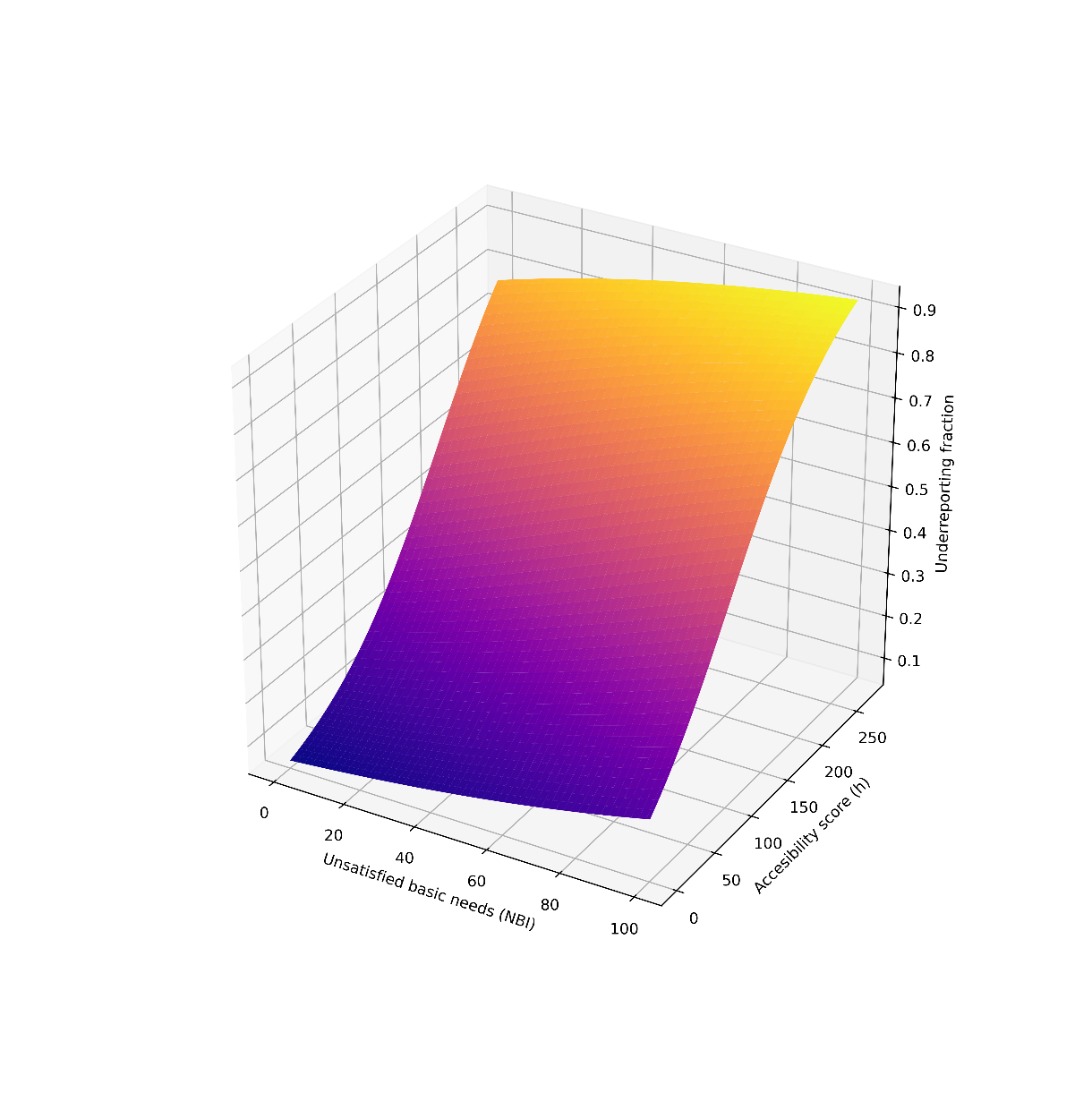
**

| **Parameter** | **5% CI** | **95% CI** |
| --- | --- | --- |
| *b_1_* | 2.66 | 3.08 |
| *b_2_* | -3.78e-02 | -6.86e-07 |
| *b_3_* | -2.51e-02 | -2.52e-04 |

***Fig S3****. Dependence of underreporting on accessibility score and poverty after model parametrization.* As we stated in the model, there is a positive correlation between both variables and underreporting, where low public health coverage and high poverty will increase underreporting fraction. Therefore, our estimation of underreporting varies between 5.68% and 37.83%. Credible intervals were obtained by computing the highest density intervals for posterior distribution.
