# Supplementary material for: A generalized framework for estimating snakebite underreporting using statistical models: A study in Colombia
Source: PLoS Negl Trop Dis. 2023 Feb 6;17(2):e0011117. doi: 10.1371/journal.pntd.0011117 (PMC9934346; doi:10.1371/journal.pntd.0011117)
Supplement: S4 Fig — We used georeferenced data of medical centers with the capacity to administrate antivenom, and we determined travel speed with maps of roads, fluvial routes, slope, and land coverage. (DOCX) [file pntd.0011117.s004.docx]

**
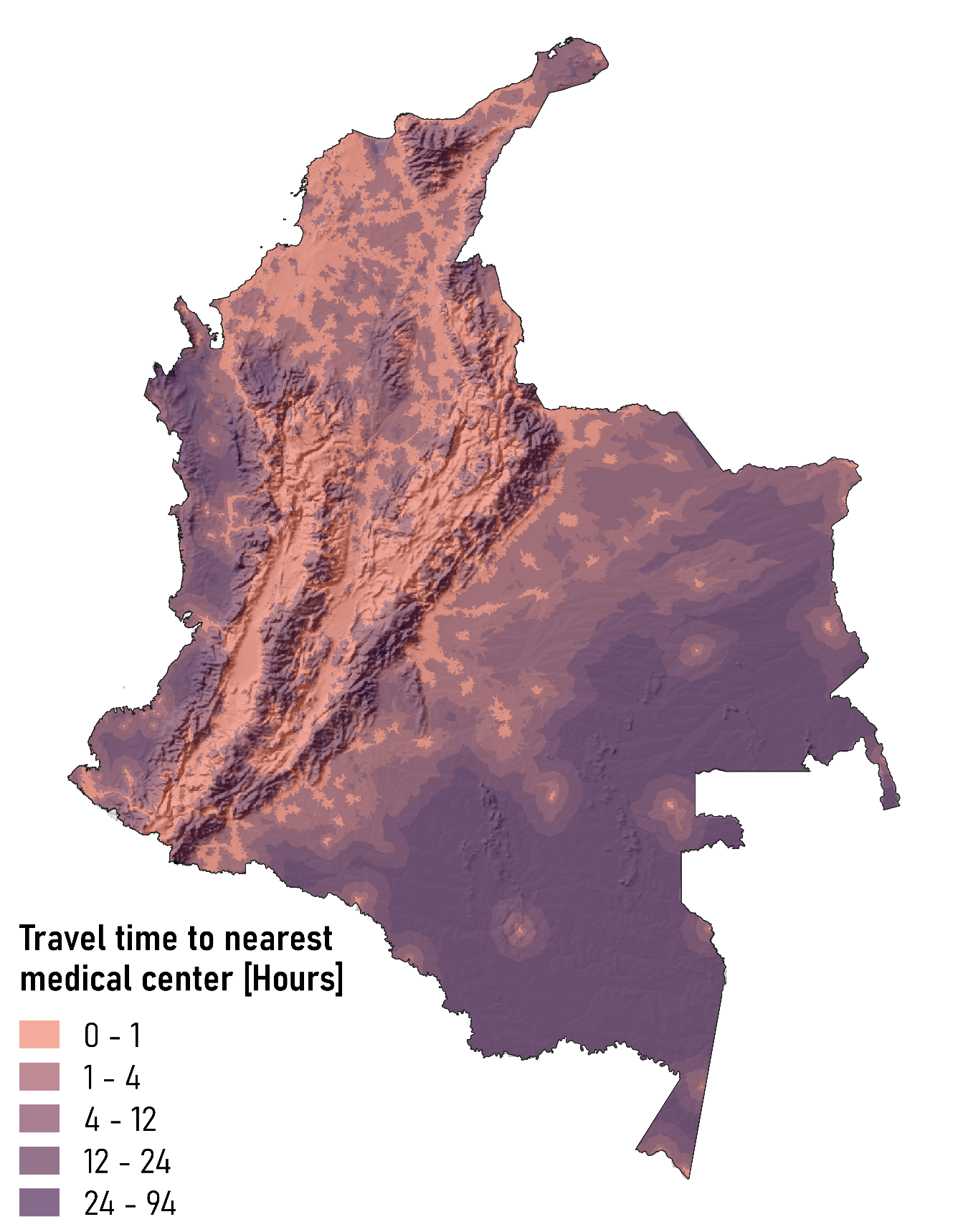
**

***Fig S4.*** *Travel time to the nearest medical center.* We used georeferenced data of medical centers with the capacity to administrate antivenom, and we determined travel speed with maps of roads, fluvial routes, slope, and land coverage. Base map of national boundaries of Colombia was obtained from DIVA-GIS free spatial data (https://www.diva-gis.org/datadown).
