# Supplementary material for: A generalized framework for estimating snakebite underreporting using statistical models: A study in Colombia
Source: PLoS Negl Trop Dis. 2023 Feb 6;17(2):e0011117. doi: 10.1371/journal.pntd.0011117 (PMC9934346; doi:10.1371/journal.pntd.0011117)
Supplement: S1 Text — (DOCX) [file pntd.0011117.s005.docx]

**S1 text.** *Maximum entropy model calibration and selection of the best distribution models*

We used the package *ENMeva*l in r to perform the niche modeling by entropy maximization. First, we defined as background a buffer around the depurated occurrence locations for each species. This buffer had a ratio of 100 km, and we randomly selected 100 points over this buffer as background records. The aim of this step is to compare the climatic conditions of each presence location with the climatic conditions of the background points during the maximum entropy algorithm. Thus, background or “pseudoabsence” data will be selected in the surroundings of each presence point. The buffer and the background locations can be seen in figure 1.


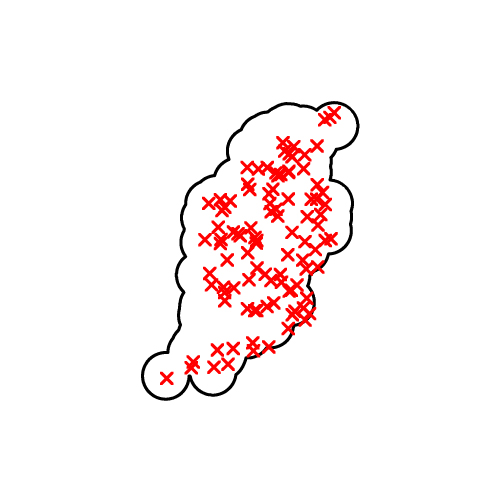

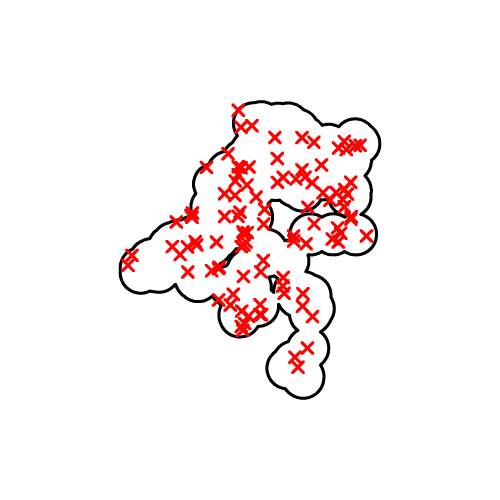


*Figure 1. Background locations selected on buffer regions surrounding presence locations.*

Then, we evaluated different models in *ENMeval* by varying feature classes (These are the potential mathematic shape of the response curves between suitability and climatic variable) and the regularization multiplier (This parameter allows to penalize over-complexed models by adjusting the error bound between the prediction and the observed data). We selected the six most used feature classes (Lineal, Lineal-Quadratic, Hinge, Lineal-Quadratic-Hinge, Lineal-Quadratic-Hinge-Product and Lineal-Quadratic-Hinge-Product-Threshold), and also we defined the regularization multiplier between 1 and 6 (1). To partition data avoiding possible spatial autocorrelation in presence records, we used the block partitioning algorithm, which selects 4 blocks in the geographic area that contains approximately the same quantity of presence records, and select 3 blocks of data to train the model and the other one to test it (2). The results of this block partitioning can be seen in figure 2.

The code for replicating this study can be found in figshare (DOI: 10.6084/m9.figshare.21779342). This specific section is found in folder Niche mod.


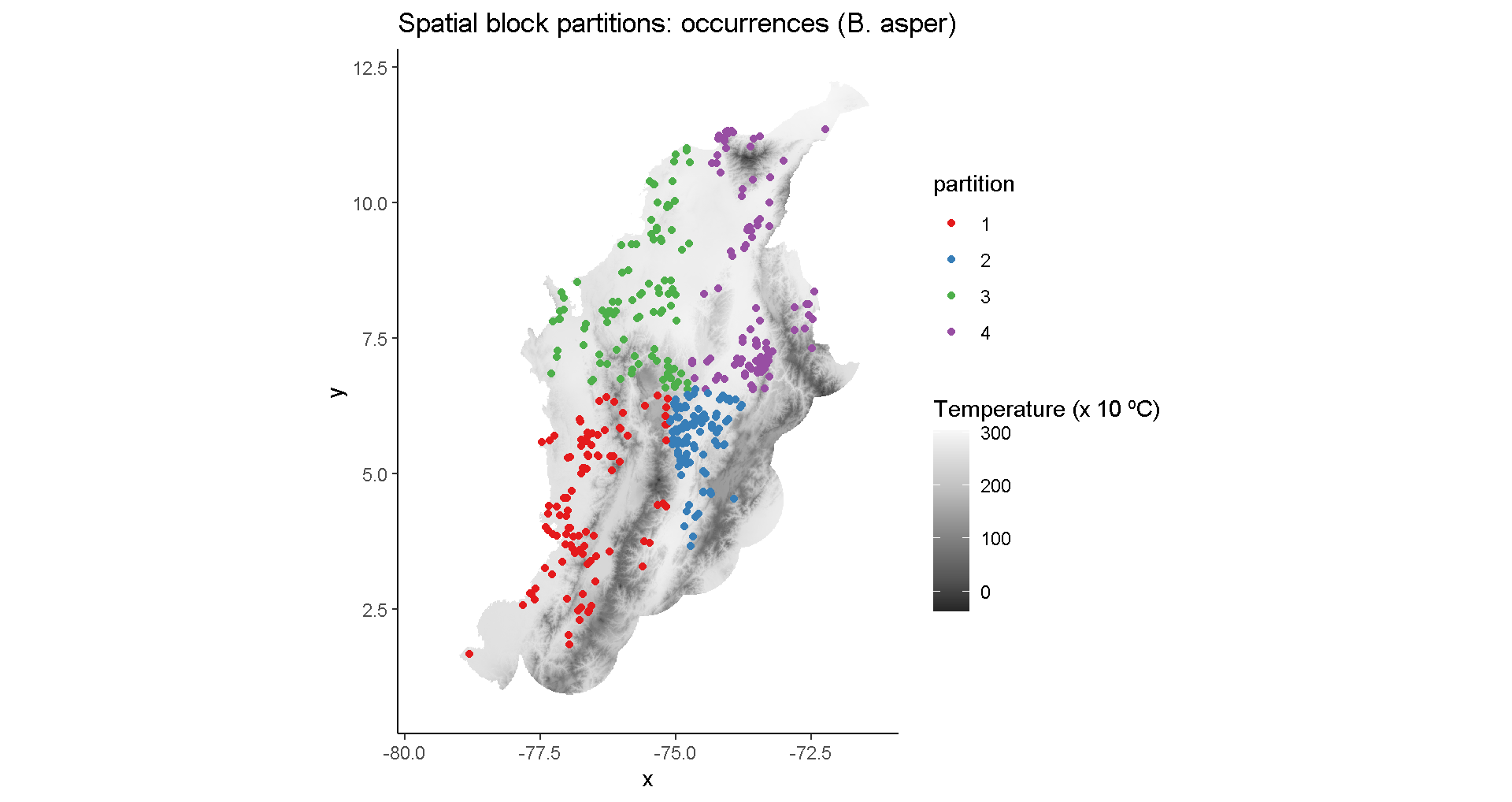

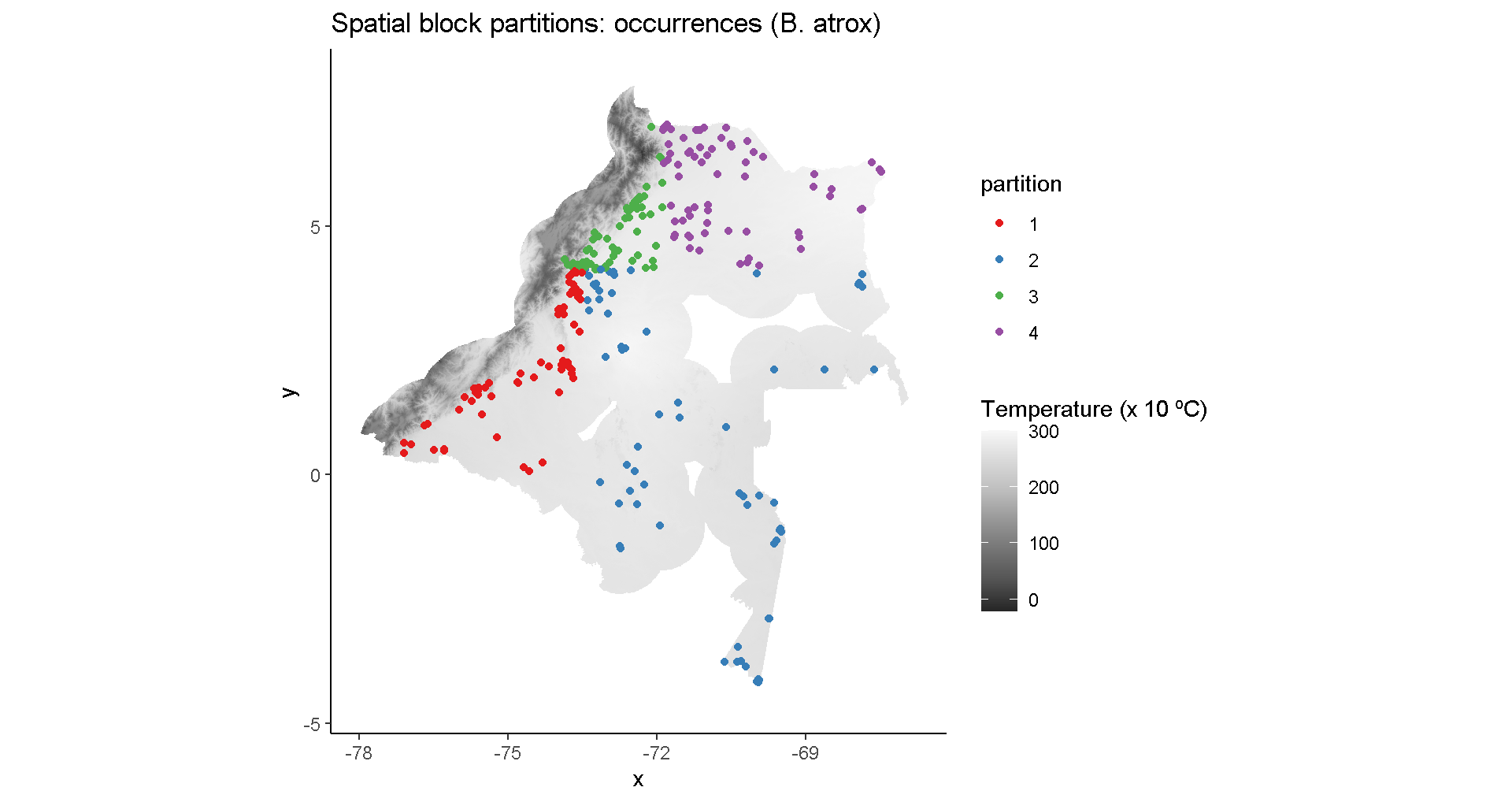


*Figure 2. Block partition of presence data for both species.* Base map of elevation of Colombia was obtained from WORLDCLIM server (https://www.worldclim.org/data/worldclim21.html).

Finally, after performing maxent modelling in *ENMeval* package for each one of the combinations between feature classes and regularization multiplier, we selected the 10 models with the lowest AICc per each species. Our habitat suitability model and its performance evaluation was performed using *dismo* and *ENMeval* packages in *R* environment (3–5). Code is available at LINK FIGSHARE.

**REFERENCES**

1. Phillips SJ, Anderson RP, Schapire RE. Maximum entropy modeling of species geographic distributions. Ecol Modell [Internet]. 2006;190(3):231–59. Available from: http://www.sciencedirect.com/science/article/pii/S030438000500267X

2. Radosavljevic A, Anderson RP. Making better Maxent models of species distributions: complexity, overfitting and evaluation. J Biogeogr [Internet]. 2014 Apr 1 [cited 2021 Oct 21];41(4):629–43. Available from: https://onlinelibrary.wiley.com/doi/full/10.1111/jbi.12227

3. Hijmans RJ, Phillips S, Leathwick J, Elith J. dismo: Species Distribution Modeling [Internet]. 2017. Available from: https://cran.r-project.org/package=dismo

4. R Development Core Team. R: A Language and Environment for Statistical Computing [Internet]. Vienna, Austria; 2008. Available from: http://www.r-project.org

5. Muscarella R, Galante PJ, Soley-Guardia M, Boria RA, Kass JM, Uriarte M, et al. ENMeval: An R package for conducting spatially independent evaluations and estimating optimal model complexity for Maxent ecological niche models . Methods Ecol Evol [Internet]. 2014 Nov 1 [cited 2021 Jun 11];5(11):1198–205. Available from: https://besjournals.onlinelibrary.wiley.com/doi/full/10.1111/2041-210X.12261
