# Supplementary material for: A generalized framework for estimating snakebite underreporting using statistical models: A study in Colombia
Source: PLoS Negl Trop Dis. 2023 Feb 6;17(2):e0011117. doi: 10.1371/journal.pntd.0011117 (PMC9934346; doi:10.1371/journal.pntd.0011117)
Supplement: S1 Table — (DOCX) [file pntd.0011117.s006.docx]

**Table S1.** *Environmental layers used for ecological niche modeling.*

| **Variable** | **Units** | **Source** |
| --- | --- | --- |
| Mean temperature of warmer quarter | °C*10 | WorldClim |
| Mean Diurnal Range | °C*10 | WorldClim |
| Isothermality | - | WorldClim |
| Annual Precipitation | mm | WorldClim |
| Precipitation of Driest Month | mm | WorldClim |
| Precipitation of Warmest Quarter | mm | WorldClim |
