# Supplementary material for: A generalized framework for estimating snakebite underreporting using statistical models: A study in Colombia
Source: PLoS Negl Trop Dis. 2023 Feb 6;17(2):e0011117. doi: 10.1371/journal.pntd.0011117 (PMC9934346; doi:10.1371/journal.pntd.0011117)
Supplement: S2 Table — (DOCX) [file pntd.0011117.s007.docx]

**Table S2.** *Travel speeds used for different land coverages.*

| **Land coverage** | **Speed [km/h]** |
| --- | --- |
| Unknown | NA |
| Shrubs: Woody plants < 5m | 3 |
| Herbaceous vegetation | 3 |
| Agricultural crops | 4 |
| Urban lands | 5 |
| Disperse vegetation: Exposed soil | 4 |
| Snow and ice | 0.5 |
| Permanent water | 0.5 |
| Flooded shrubs and herbaceous vegetation | 0.5 |
| Moss and Lichen | 4 |
| Closed forest | 2 |
| Open forest | 2 |
| Water bodies | 0.5 |
| Primary road | 50 |
| Secondary road | 30 |
| Tertiary road | 15 |
| Trunk | 60 |
| Road | 15 |
| Unclassified | 15 |
| Track | 10 |
| Rivers with fluvial transportation | 8 |
