# Supplementary material for: A generalized framework for estimating snakebite underreporting using statistical models: A study in Colombia
Source: PLoS Negl Trop Dis. 2023 Feb 6;17(2):e0011117. doi: 10.1371/journal.pntd.0011117 (PMC9934346; doi:10.1371/journal.pntd.0011117)
Supplement: S3 Table — (DOCX) [file pntd.0011117.s008.docx]

**Table S3.** *Variable importance for both species after niche modeling calibration and selection*

| **Bioclimatic variable** | ***Bothrops asper*** | ***Bothrops atrox*** |
| --- | --- | --- |
| **B10** | 99.56 | 37.93 |
| **B2** | 0.00 | 0.09 |
| **B3** | 0.06 | 5.72 |
| **B12** | 0.36 | 2.25 |
| **B14** | 0.02 | 45.60 |
| **B18** | 0.00 | 8.40 |
