# Supplementary material for: A generalized framework for estimating snakebite underreporting using statistical models: A study in Colombia
Source: PLoS Negl Trop Dis. 2023 Feb 6;17(2):e0011117. doi: 10.1371/journal.pntd.0011117 (PMC9934346; doi:10.1371/journal.pntd.0011117)
Supplement: Attachment — Submitted filename: PNTD-D-22-00894.pdf [file pntd.0011117.s009.pdf]

PLOS Neglected Tropical Diseases

A generalized framework to estimate snakebite underreporting using statistical models: A study in Colombia

--Manuscript Draft--

**Manuscript Number:**	PNTD-D-22-00894
**Full Title:**	A generalized framework to estimate snakebite underreporting using statistical models: A study in Colombia
**Short Title:**	Statistical model to estimate snakebite underreporting: A study in Colombia
**Article Type:**	Research Article
**Keywords:**	Snakebite; Underreporting; Statistical model; Bayesian inference; Incidence estimation
**Abstract:**	Background<br>Snakebite envenoming is a Neglected Tropical Disease affecting mainly deprived population, and its burden is normally underestimated because patients prefer to seek for traditional medicine. Thus, applying strategies to optimize disease' management is difficult. We propose a framework that estimates total snakebite incidence at fine political scale.<br>Methodology/Principal findings<br>First, we produced snakebite fine-scale risk maps based on venomous snakes' distribution. Then, we proposed a generalized mixed effect model that estimates total incidence based on risk maps, poverty, and travel time to the nearest medical center. Finally, we calibrated our model with snakebite reported data in Colombia from 2010 to 2019 using Markov chain Monte Carlo. Our results suggest that 10.3% of total snakebite cases (538.3 yearly envenomings) are not reported and do not seek medical attention, and that the Orinoco and Amazonian regions share high risk and underreporting. We also found that variables such as climatic seasonality influences venomous snake suitability rather than absolute temperature or rainfall.<br>Conclusions/Significance<br>Our framework can estimate snakebite underreporting by using base data of snakebite incidence and surveillance, presence locations for most important venomous snake species, and online available information of census, poverty, climate, land cover, roads, and geolocations of medical centers. Thus, our algorithm can be used in other countries to estimate total incidence and to improve disease management, but it will not replace a correct surveillance system that must be a priority in countries affected by this problem.
**Additional Information:**	
**Question**	**Response**
**Financial Disclosure**<br><br>Enter a financial disclosure statement that describes the sources of funding for the work included in this submission. Review the submission guidelines for detailed requirements. View published research articles from *PLOS NTDs* for specific examples.<br><br>This statement is required for submission and **will appear in the published article** if the submission is accepted. Please make sure it is accurate.	This study was partially supported by: Minciencias, Colombia (https://www.minciencias.gov.co/), Application 727 for doctoral student to CBV, and Universidad de los Andes, Colombia (https://uniandes.edu.co/), Funding program for doctoral students, awarded to CBV. The funders had no role in study design, data collection and analysis, decision to publish, or preparation of the manuscript.

Powered by Editorial Manager® and ProduXion Manager® from Aries Systems Corporation

**Unfunded studies**<br><br>Enter: *The author(s) received no specific funding for this work.*<br><br>**Funded studies**<br><br>Enter a statement with the following details:<br>• Initials of the authors who received each award<br>• Grant numbers awarded to each author<br>• The full name of each funder<br>• URL of each funder website<br>• Did the sponsors or funders play any role in the study design, data collection and analysis, decision to publish, or preparation of the manuscript?<br>• **NO** - Include this sentence at the end of your statement: *The funders had no role in study design, data collection and analysis, decision to publish, or preparation of the manuscript.*<br>• **YES** - Specify the role(s) played.<br><br>* typeset	
**Competing Interests**<br><br>Use the instructions below to enter a competing interest statement for this submission. On behalf of all authors, disclose any competing interests that could be perceived to bias this work—acknowledging all financial support and any other relevant financial or non-financial competing interests.<br><br>This statement **will appear in the published article** if the submission is accepted. Please make sure it is accurate. View published research articles from *PLOS NTDs* for specific examples.	The authors have declared that no competing interests exist.

**NO authors have competing interests**<br><br>Enter: *The authors have declared that no competing interests exist.*<br><br>**Authors with competing interests**<br><br>Enter competing interest details beginning with this statement:<br><br>*I have read the journal's policy and the authors of this manuscript have the following competing interests: [insert competing interests here]*<br><br>* typeset<br><br>This statement is required for submission and **will appear in the published article** if the submission is accepted. Please make sure it is accurate and that any funding sources listed in your Funding Information later in the submission form are also declared in your Financial Disclosure statement.	
**Data Availability**<br><br>Authors are required to make all data underlying the findings described fully available, without restriction, and from the time of publication. PLOS allows rare exceptions to address legal and ethical concerns. See the PLOS Data Policy and FAQ for detailed information.	Yes - all data are fully available without restriction

A Data Availability Statement describing where the data can be found is required at submission. Your answers to this question constitute the Data Availability Statement and **will be published in the article**, if accepted.<br><br>**Important:** Stating 'data available on request from the author' is not sufficient. If your data are only available upon request, select 'No' for the first question and explain your exceptional situation in the text box.<br><br>Do the authors confirm that all data underlying the findings described in their manuscript are fully available without restriction?	
**Describe where the data may be found in full sentences. If you are copying our sample text, replace any instances of XXX with the appropriate details.**<br><br>• If the data are **held or will be held in a public repository**, include URLs, accession numbers or DOIs. If this information will only be available after acceptance, indicate this by ticking the box below. For example: *All XXX files are available from the XXX database (accession number(s) XXX, XXX.*).<br>• If the data are all contained **within the manuscript and/or Supporting Information files**, enter the following: *All relevant data are within the manuscript and its Supporting Information files.*<br>• If neither of these applies but you are able to provide **details of access elsewhere**, with or without limitations, please do so. For example:	All databases and code requiered to replicate our results will be held at a repository recommended by the editorial staff.

Data cannot be shared publicly because of [XXX]. Data are available from the XXX Institutional Data Access / Ethics Committee (contact via XXX) for researchers who meet the criteria for access to confidential data.<br><br>*The data underlying the results presented in the study are available from (include the name of the third party and contact information or URL).*<br>• This text is appropriate if the data are owned by a third party and authors do not have permission to share the data.<br><br>* typeset	
Additional data availability information:	Tick here if the URLs/accession numbers/DOIs will be available only after acceptance of the manuscript for publication so that we can ensure their inclusion before publication.

Powered by Editorial Manager® and ProduXion Manager® from Aries Systems Corporation

Manuscript
Click here to access/download;Manuscript;Draft Final.docx

Full title: A generalized framework to estimate snakebite underreporting using statistical models: A study in Colombia

Short title: Statistical model to estimate snakebite underreporting: A study in Colombia

Carlos Bravo-Vega[1*], Camila Renjifo-Ibañez[2+], Mauricio Santos-Vega[1,3+], Leonardo Jose León Nuñez[4], Teddy Angarita-Sierra[5], Juan Manuel Cordovez[1].

[1] Grupo de investigación en Biología Matemática y Computacional (BIOMAC), Departamento Ingeniería Biomédica, Universidad de los Andes, Bogotá, Colombia.

[2] Corporación colombiana de investigación agropecuaria, AGROSAVIA, Bogotá, Colombia.

[3] Facultad de Medicina, Universidad de los Andes, Bogotá, Colombia.

[4] Observatorio de Salud Pública y epidemiología "José Felix Patiño", Universidad de los Andes, Bogotá, Colombia.

[5] Grupo de investigación en animales ponzoñosos y sus venenos, Dirección de Producción, Instituto Nacional de Salud, Bogotá, Colombia.

+ These authors contributed equally

* Correspondence author

ABSTRACT

Background

Snakebite envenoming is a Neglected Tropical Disease affecting mainly deprived population, and its burden is normally underestimated because patients prefer to seek for traditional medicine. [illegible]hus, applying strategies to optimize disease' management is difficult. We propose a framework that estimates total snakebite incidence at fine political scale.

Methodology/Principal findings

First, we produced snakebite fine-scale risk maps based on venomous snakes' distribution. Then, we proposed a generalized mixed effect model that estimates total incidence based on risk maps, poverty, and travel time to the nearest medical center. Finally, we calibrated our model with snakebite reported data in Colombia from 2010 to 2019 using Markov chain Monte Carlo. Our results suggest that 10.3% of total snakebite cases (538.3 yearly envenomings) are not reported and do not seek medical attention, and that the Orinoco and Amazonian regions share high risk and underreporting. We also found that variables such as climatic seasonality influences venomous snake suitability rather than absolute temperature or rainfall.

Conclusions/Significance

Our framework can estimate snakebite underreporting by using base data of snakebite incidence and surveillance, presence locations for most important venomous snake species, and online available information of census, poverty, climate, land cover, roads, and geolocations of medical centers. Thus, our algorithm can be used in other countries to estimate total incidence and to improve disease management, but it will not replace a correct surveillance system that must be a priority in countries affected by this problem.

AUTHOR SUMMARY

Snakebite envenoming is a challenging neglected tropical disease, where planning is difficult because of incidence underestimation. This underreporting is caused by the low coverage of health centers in tropical countries and patients' preference for seeking traditional medicine. Currently, there are mathematical approaches that explain snakebite incidence by parameter adjustment, but these approximations have not been expanded to estimate envenomings that are not reported to medical centers. Our study proposes a statistical framework to estimate snakebite underreporting. To our knowledge, this is the first time that cases that are not being attended by health care can be estimated computationally, which highly decreases time and recourses compared with field epidemiological studies. Our modelling scheme can be used to give a quick estimation of the spatial heterogeneity of snakebite underreporting, which can be used to detect areas in need of accessibility improvement. Also, it helps to determine the expected number of cases, which can be used to increase community empowerment into public health and to determine the real demand of antivenom. We want to stress that this tool can be extrapolated to different countries, but it will never substitute a correct surveillance system.

INTRODUCTION

Snakebite envenoming is a Neglected Tropical Disease (NTD) characterized by its high mortality and morbidity rates (1–4). The most effective treatment is the administration of antivenom, which is composed of a mixture of antibodies (5). Although antivenom is life-saving, most patients affected by a snakebite live in remote rural areas where it is not readily available (6,7). Envenomation episodes in rural areas often result in patients seeking traditional healers instead of antivenom

therapy (4,8–11). This situation poses a challenge in data collection, which usually does not account for the number of envenoming cases (12,13). As a global effort to reduce snakebite mortality by 50% in 2030, the world health organization proposed a global strategy where one aim corresponds with strengthening surveillance systems, improving antivenom availability, and including this NTD into public health scope. Indeed, the first step to fulfill this objective is to generate reliable estimates of snakebite burden, where disease eco-epidemiology can help pinpointing overlooked vulnerable populations (14,15).

Estimating disease burden by performing cross-sectional studies requires resources that are not readily available (16–18). Therefore, it is necessary to study the natural history of the species involved in the disease and then perform mathematical modeling to estimate epidemiological parameters (19–22). This biological knowledge becomes critical in megadiverse countries like Colombia, where approximately 319 species of snakes can be found, but only 52 represent a potential risk for humans. Out of these 52 species, 21 belong to the *Viperidae* family and 31 belong to the *Elapidae* family (23–25). These venomous snakes are distributed in most of the territory, and human–snake encounters occur frequently (26–28). The most medically important venomous snake species in the country are *Bothrops asper* and *B. atrox*, which are responsible for most of the envenomings (some studies suggest that it could be as much as 80%) (25,28–33). Previous studies for Colombia have proposed that the species range for *B. asper* is the Caribbean, Pacific coast, and the inter-Andean valleys. On the contrary, the species range of *B. atrox* ranges from the Orinoco and Amazonian regions (25). However, their

distribution and association with environmental variables are largely absent in scientific studies (34,35).

Snakebite envenoming usually lacks reliable and long-term data accounting for the number of cases in each region in a country. In Colombia, snakebite cases treated in medical centers are reported to Sistema Nacional de Vigilancia en Salud Pública (SIVIGILA), which monitors and collects public health data the country (29,36). In 2004, the report of snakebite cases became mandatory for each medical center, but later in 2008 the report became individual for each patient (37). The only snakebite incidence report prior 2004 was from 1975 to 1999, with an average of 0.18 envenoming's per 100.000 inhabitants nationwide (70.8 cases per year) (29). In 2005 SIVIGILA reported 5.03 envenoming's per 100.000 inhabitants (2161 cases per year), and later, in 2016 the reported incidence was of 9.6 envenoming's per 100.000 inhabitants (4704 cases per year). This sudden increase of cases seems to be associated with implementing the new reporting system rather than a change in the dynamics of snakebite (36,38).

Nevertheless, the total number of reported cases is still expected to be less than the total cases due to the complexities of data collection in the territory (4,6,18,27,29,39). In Colombia, antivenom cost is borne by the government, but its distribution usually does not reach the needs areas (12,40). Therefore, estimating disease burden through mathematical approaches could help determine the total number of snakebite cases in the country and the real demand for antivenom, thus helping to fulfill WHO global strategy (10,14,16,41,42).

Our paper focuses on generating and algorithm to estimate underreporting by using as a case study Colombia. To calibrate this model, we first developed a risk estimator based on the distributions of *Bothrops asper* and *B. atrox*, and we tested its performance with snakebite public health records. Second, we used a generalized mixed effect model, which incorporates the risk estimator as a proxy to estimate total snakebite cases, and povert[illegible]data with an accessibility index to estimate the reported fraction of these total cases. Third, we calibrated our model with Spatio-temporal data of snakebite in Colombia from 2010 to 2019 by using the Markov-Chain-Monte-Carlo (MCMC) algorithm in NIMBLE r-package (43), and finally, we produced maps of underreporting and total incidence that report the real burden of this NTD.

METHODOLOGY

We followed three steps to generate and calibrate our model: First, we built an envenoming risk score map based on snake distributions (obtained through ecological niche modeling) and the law of mass action. Second, we calculated an accessibility score that reflects the struggle to reach a medical center. Finally, we developed and parametrized a mixed mathematical model to estimate underreporting.

Snakebite's envenoming risk score map

The envenoming snakebite risk is mainly associated with the presence of venomous snakes. Therefore, we developed an envenoming risk score incorporating the ecological niche modeling scores for the two species (*SNMS*, see below). This risk

score is based on the law of mass action, which states that envenoming will be the result of encounters between humans and snakes, and it will be proportional to the multiplication of their abundances (16):

$$I = \alpha \times S \times P \quad (3)$$

In Eq. 3, *I* is the snakebite incidence, *S* is the snake's abundance, *P* is the rural population, and α the contact rate between both populations. Dividing the equation by *P*, approximating *S* to *SNMS*, and using a logarithmic transformation on the reported risk to linearize its relationship with *SNMS*, we obtain our estimation of risk (Eq. 4):

$$Ln\left(\frac{I}{P}\right) \approx Snakebite\ risk \approx a \times SNMS \quad (4)$$

Snake presence data

We selected *Bothrops asper* and *B. atrox* as study species because of their wide range, ability to adapt to human intervention, and responsibility for most snakebites in Latin America and Colombia (25,27–29,32,33,35,44,45). First, we built a database of presence records using georeferenced samples obtained from natural history museums in Colombia (See acknowledgments), where duplicated records were eliminated for each species. Then, to homogenize the spatial data, we used a grid of 0.3° resolution for *B. asper* and 0.7° resolution for *B. atrox* to select only one presence point per square-grid (View Fig S1). Finally, we removed records above 1900 m.a.s.l. for *B. asper* and 1500 m.a.s.l. for *B. atrox* because these are the maximum altitude thresholds known for their distribution in South America (25,31).

Our initial snake presence database had a total of 636 records for *B. asper*, and 374 records for *B. atrox*. After data depuration, our final dataset had 104 and 65 records for each species respectively.

Environmental layers

We used Bio-climate variables from the WorldClim database server (http://www.worldclim.org) (46) for the current conditions in a resolution of approximately 1 km x 1 km. We removed collinear environmental layers with the package *Virtual Species* in R by using a threshold of 0.9 over the Pearson's R correlation coefficient (47–49). The selected Bioclimatic variables are shown in Table S1.

Envenoming risk map estimation and validation

We predicted species habitat suitability using a maximum entropy algorithm because we used only presence data (50). After performing the algorithm described in S1 text, we generated a set of maps representing the ten best distribution models for each species over each species range to produce habitat suitability maps using a cloglog output format (51). Then, we removed areas above known altitude thresholds for both species (25,31). Finally, given that both species are not sympatric (25,31), we combined all the possible permutations of these ten predictions to obtain 100 maps. These score maps are the snake niche modeling score per pixel (*SNMS)*.

To select the best *SNMS** map to be proposed as our final envenoming risk score map (*SNMS*), we computed the average value of each *SNMS** map for each department, and we compared it with the logarithm of reported snakebite risk

(snakebite incidence per 100.000 persons). To compute reported risk, we averaged SIVIGILA reported incidence (which correspond to cases that received medical attention) between 2010 and 2019, and we obtained rural population estimates from the National Administrative Department of Statistics (DANE).

To compare *SNMS** with reported risk, we used a type II linear regression by using ordinary least squares (View Eq. 4) (52). Finally, we selected the *SNMS** map with the highest correlation with reported risk as our final envenoming risk score map (*SNMS)*.

Computation of the accessibility score

We assumed that the distance to nearest medical center is the only factor affecting antivenom accessibility. Other aspects that can contribute to accessibility are the availability of serum or the presence of trained personnel (33,53,54). Unfortunately, we did not have access to information about these factors. Hence, we first created a combined map of travel speed by using maps of: i) Roads and type of road from the World Food Programme and Open Street maps (55), ii) Land coverage from the Food and Agriculture Organization of the United Nations (56), iii) Fluvial transport from the Agustin Codazzi institute (57) and *iv)* Geographic slope map computed using *raster* package in R environment (47,58) and an altitude map from the server WorldClim (46). Then, we computed the minimum travel time for each location to the nearest medical center based on the travel speed map obtained by merging input maps. We used a georeferenced dataset of clinics, hospitals, and health centers in the 2016 Geostatistical National Framework published by the DANE (59). This

algorithm was performed using the package gDistance in R (60), and the output is a map of minimum travel time to the nearest medical center.

Statistical modelling of snakebite underreporting

Model Description

We based our mathematical model in the hierarchical model proposed by (61). First, to model the *real* incidence of snakebite, we used the law of mass action because it correctly represents the real incidence of snakebite (16). By using Eq. 4, and writing the model as a generalized lineal model, we can state the following:

$$Ln\left(\frac{\bar{I}_i}{P_i}\right) = \alpha_1 + \alpha_2 SNMS_i + \theta_i \tag{5}$$

Where $\bar{I}$ is the real incidence of snakebite, and α_1 and α_2 are the intersect and the slope of the generalized lineal model respectively, θ accounts for a normal random noise, and sub-index *i* denotes the geographical scale, in our case municipality. Assuming the population as an offset we can re-write the model as (Eq. 6):

$$Ln(\bar{I}_i) = Ln(P_i) + \alpha_1 + \alpha_2 SNMS_i + \theta_i \tag{6}$$

Then, we defined the reporting of snakebite incidence as a counting Poisson process, where the mean is the real incidence (Eq. 6) times a reporting fraction π_i (61). So, reported snakebite incidence ${I^r}_i$ can be modelled as:

$${I^r}_i = Poisson(\lambda = \pi_i \bar{I}_i) \tag{7}$$

To estimate the reporting fraction we assumed that it will depend on the proposed accessibility score and a poverty index (2,13,39,54,62,63). We used the unsatisfied basic needs index as the poverty index, and we defined the accessibility score for each municipality as the geographic-averaged travel time (64). By assuming a general linear dependence, the model for reporting fraction is the following:

$$ln\left(\frac{\pi_i}{1-\pi_i}\right) = b_1 + b_2 AS_i + b_3 NBI_i \quad (8)$$

We applied the logit function to the reporting fraction π_i to guarantee that this fraction will be between 0 and 1. In this part of the model, b_1, b_2, and b_3 are parameters, AS is the accessibility score, and NBI is the poverty index. Thus, the complete model to estimate *real* incidence is the following (Eq. 9):

$$\begin{gathered} {I^r}_i = Poisson(\lambda = \pi_i \bar{I}_i) \\ Ln(\bar{I}_i) = Ln(P_i) + \alpha_1 + \alpha_2 SNMS_i + \theta_i \\ ln\left(\frac{\pi_i}{1-\pi_i}\right) = b_1 + b_2 AS_i + b_3 NBI_i \end{gathered} \quad (9)$$

Model calibration

To estimate the model parameters we used the MCMC algorithm, which uses a Bayesian approach to fit the model to data (43,65) (prior distributions are shown in Fig S2). First, the posterior distributions for the parameters were estimated by using the NIMBLE package in R environment (43,47). Next, we used an automated factor slice sampler (AFSS) to sample the parameters, and then we used 4 chains from different initial conditions to ensure a global optimal for the convergence. We did a

total of 4.7 million iterations, where the first 57% were discarded as a burn-in, and we saved the following 43% iterations to check convergence by the Gelman-Rubin diagnostic. We used a threshold of non-convergence for this index at 1.1, where values above this threshold indicate non-convergence (66,67).

RESULTS

Venomous snakes' distribution

After determining *SNMS*, the selected model for *B. asper* had a train data AUC of 0.75, a train data CBI of 0.847, and the minimum AICc of all the models. For *B. atrox* the statistics were 0.65 (AUC), 0.76 (CBI), and the difference between its AICc and the model with the minimum AICc was 6.8. The most suitable areas for *B. asper* are the lowlands of the Middle Magdalena Basin, South Pacific region, and regions of the Caribbean Coast, while the lowest suitability was in the extremely dry areas of the northeast the country (La Guajira department). In the case of *B. atrox* the most suitable areas were located on the lowlands of the Orinoco and Amazonian regions, being suitability relatively constant, while the lowest suitability was on the piedmont of the eastern Andes (View Fig 1).

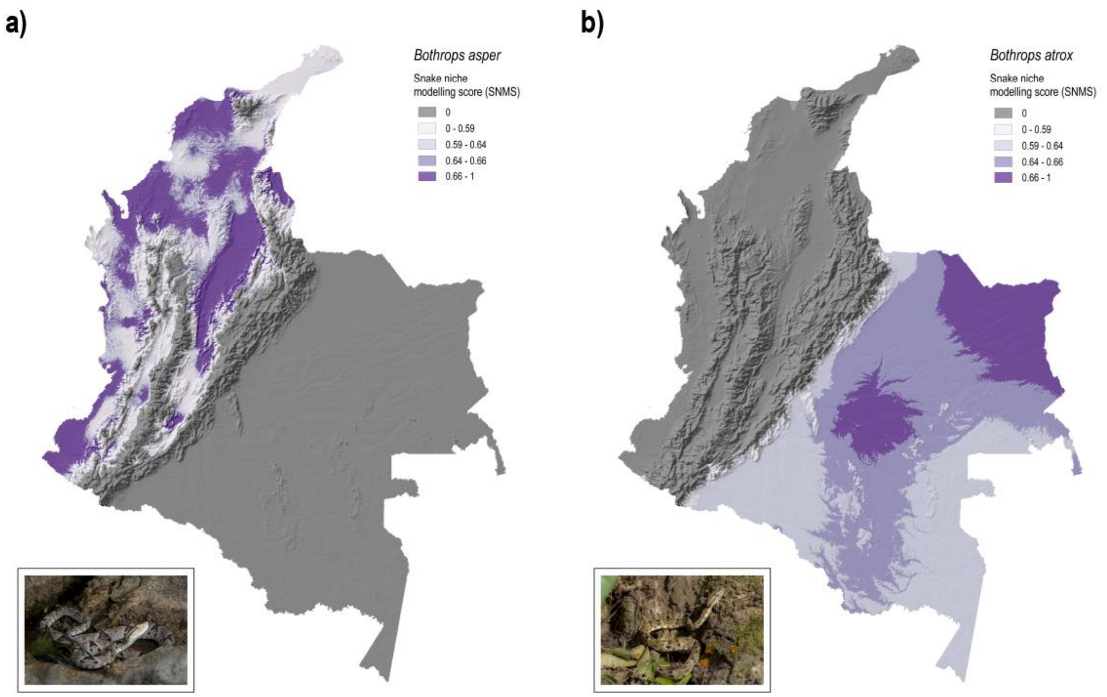

Fig 1. *Snake niche modeling score (SNMS).* Light purple areas mean 0 niche modeling scores, while light grey areas are outside each species range. Dark purple areas are high suitability areas. Both maps were produced using open-source Geographic Information System Quantum GIS (QGIS). *a) Snake niche modeling score for Bothrops asper.* Suitable areas are in the lowlands of the middle Magdalena basin, south Pacific region, and regions of the Caribbean Coast, while the lowest suitability was in the extremely dry areas of the country's northeast (la Guajira department). Note the lack of presence locations in the south of the Pacific and in the lowlands located in the south of the Caribbean coast, where is difficult to sample because of social conflict (Fig S1). *b) Snake niche modeling score for B. atrox.* Suitable areas are in the lowlands of the Orinoco and Amazonian basin, while non-suitable areas are located close to the piedmont. Note the low sampling effort in regions of the distribution of the snake, where several pristine and isolated forests are located, and data is absent (Fig S1).

Envenoming risk score

The logarithm of SIVIGILA' reported risk had a significant linear correlation with *SNMS* (Pearson's correlation coefficient: 0.77, p-value < 0.001, View Fig 2). This association suggest that the main driver of snakebite risk is the abundance or habitat suitability for venomous snakes. In addition, we found a group of high-risk departments where *Bothrops atrox* is distributed.

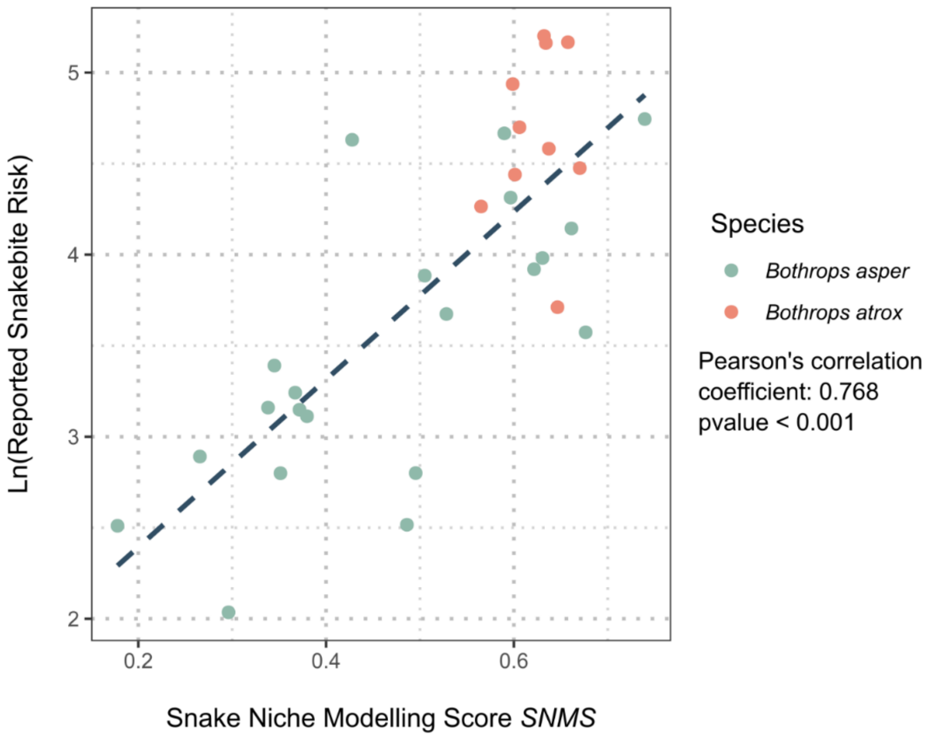

Fig 2. *Linear regression between the logarithm of SIVIGLIA (2010-2015) reported risk and envenoming risk score (SNMS) at a departmental scale.* Y-axis is the logarithm of SIVIGILA's reported risk (Envenomings per year per 100.000 persons), and X-axis shows the average envenoming risk score per department. Each point corresponds with a department, and red circles denote departments where *Bothrops asper* is distributed while blue circles are departments where *B. atrox* is found. No[illegible]he linear relationship between the two variables, which was quantified with a Pearson's correlation coefficient of 0.768 (p-

value < 0.001). Note that departments with *B. atrox* make a group characterized by high incidence and high envenoming risk score.

Underreporting estimation

The maximum index for the Gelman-Rubin diagnostic was 1.05 for α_1, and the multivariate index was 1.02 (66,67), indicating convergence. Prior and posterior distributions for model parameters are shown in Fig S2. We estimated an average number of snakebite cases of 5227.6 events per year, whereas SIVIGILA only reported an average of 4689.3 yearly events. As a result, we estimated that 538.3 cases are not reported each year, corresponding to 10.3% of the total cases. The geographic distribution of reported cases and our estimation of total cases can be seen in Fig 3. Antioquia reported most cases, which corresponds with an average of 683.4 yearly cases (Fig 3.a). Thus, each year the number of cases that do not get medical attention in the country is similar to the reported cases of the most affected department. Interestingly, departments where *Bothrops asper* is present report most snakebite cases, but the departments with *B. atrox* are riskier (Fig 3).

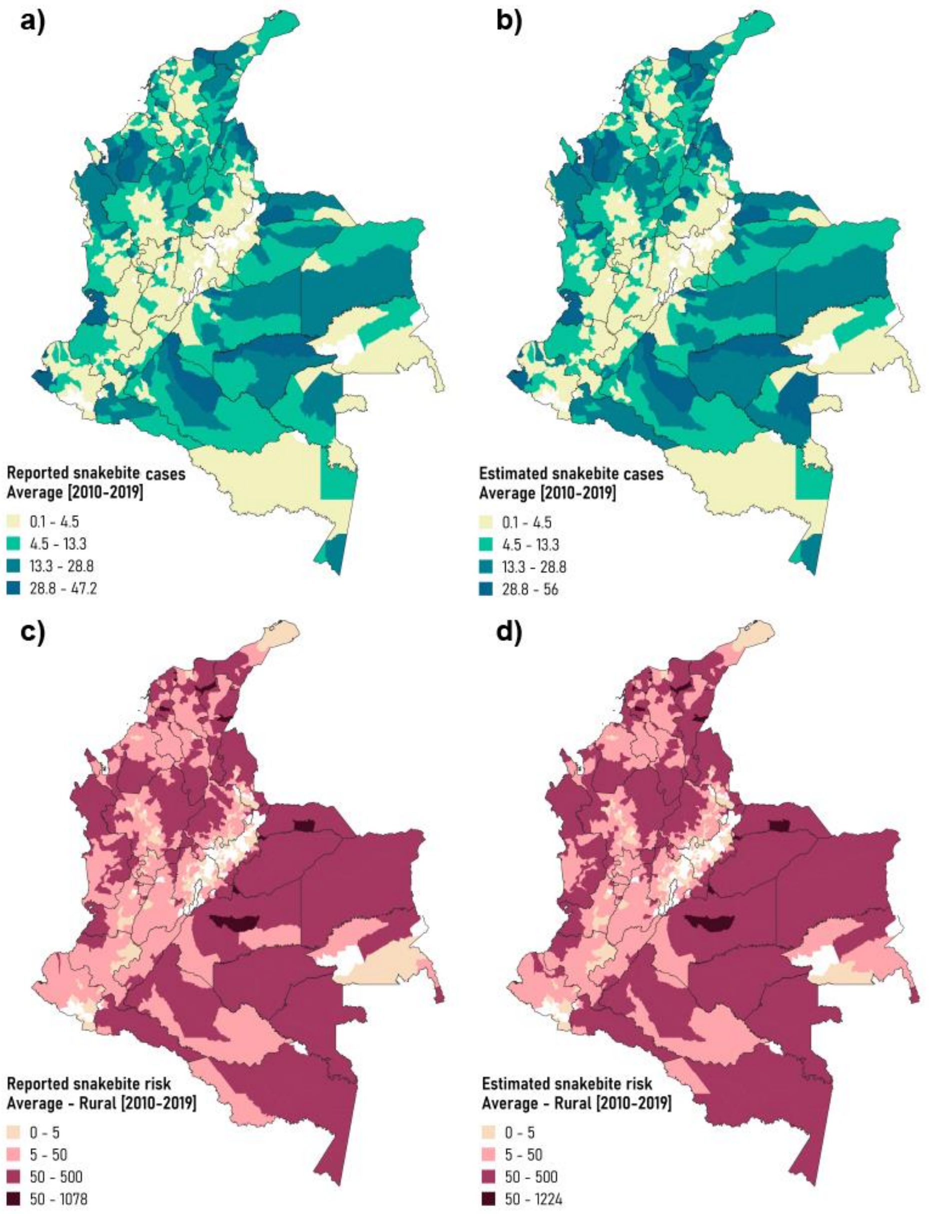

Fig 3. *Reported and estimated snakebite incidence and risk at a municipality scale. Estimates were done with the calibrated model after parameters estimation by MCMC. a) Yearly reported SIVIGILA cases (2010-2019).* Note that the spatial distribution of the cases is heterogeneous, where the department that reports most envenoming's is in Anti[illegible]ia. b) Yearly estimated total cases. Note that most municipalities increased in the scale of cases, indicating that underreporting affects most of the countr[illegible]) Yearly reported SIVIGILA

snakebite risk. Areas with higher risk are in the Orinoco and Amazonian areas, where *Bothrops atrox* is distributed. In addition, the riskiest departments are Casanare, Guaviare and Vaupes. d) Yearly estimated total snakebite risk. Most of the country have more risk than the reported ones, where departments as Amazonas and Arauca are now located in the highest risk group of departments. On the other hand, departments with *B. asper* as Cesar, Atlantico, and Norte de Santander are now the riskiest departments with this species.

Our underreporting estimates vary between 5.7% and 38.7% of total cases (Fig S3 shows the relationship between underreporting, travel time, and unsatisfied basic needs). Areas located at the eastern and southern part of the Amazonian and Orinoco regions are the most affected by underreporting, sharing the highest snakebite risk (Fig 4, Fig 3.c-d). Moreover, the Pacific and northern Caribbean coast also have a high underreporting, where these areas share high poverty and poor vial infrastructure (Fig 4)(4,39,42,64,68,69).

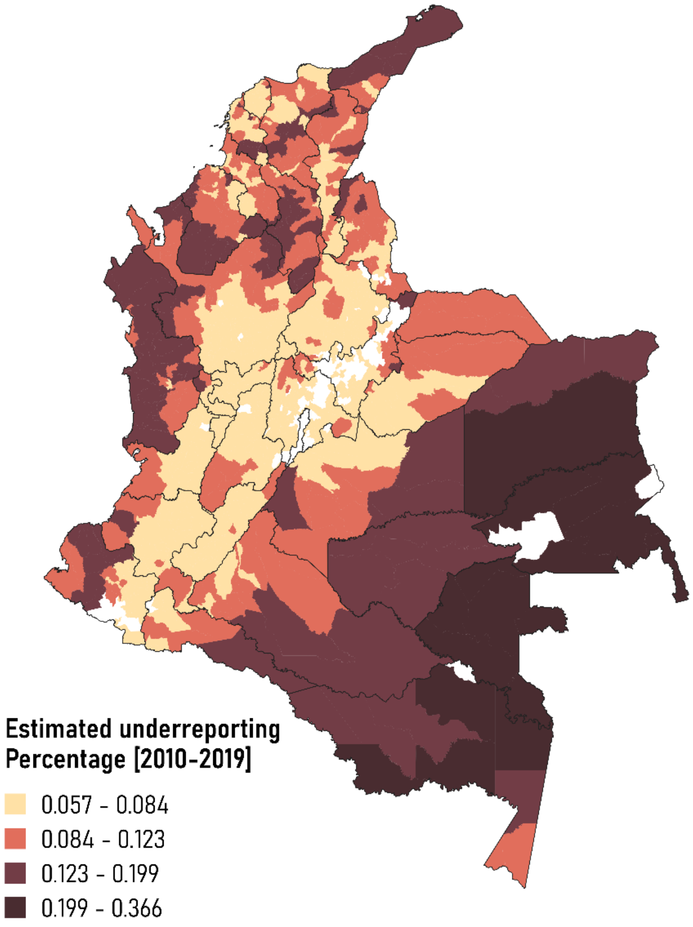

Fig 4*. Estimated snakebite underreporting.* The areas most affected by underreporting are the southeastern part of Colombia, where *Bothrops atrox* is distributed. Note that the pacific and northern Caribbean coast region also suffers from high underreporting.

DISCUSSION

We constructed a mathematical model that estimates snakebite incidence underreporting at fine political scale. Before exploring the performance and limitations of this model, we want to define the inputs required for its application: i) A set of yearly incidence data at the finest administrative level, during at least five years. Our model estimates snakebite underreporting given a set of underreported

data, but it does not estimate incidence without data. Some modifications can be done to perform a less robust estimation in areas without data, as it was discussed in (16); ii) Estimates of distribution for the most relevant venomous snake species, done by performing fieldwork (16), or by environmental niche modeling; iii) Human population datasets with the same temporal and administrative scale as the incidence data; iv) Data of main roads and fluvial routes, which can be generated by open-source datasets such as Google Maps or from ministries of transport; v) Climatic data, available in open-source servers such as WordClim or TerraClimate (46,70); vi) Geo-referenced locations of medical centers with the capacity to deal with a snakebite; and vii) A poverty estimator at the same spatial resolution than incidence and population data. These data are available for several countries, where snakebite burden can be approximated quickly with our approach.

Risk estimation validity

The estimated distribution for both species (Fig 1) seems adequate since our algorithm performance statistics (AUC and BIC) are greater than 0.5, and similar to reported values for successful predictions for different NTDs (19–21,71,72). Given that we selected models based on AICc values, we validated them using snakebite incidence, and same algorithm has been used successfully on other snakebite studies, we can rely on our distribution maps (Fig 2) (19,20).

We found that both species habitat suitability is higher on humid lowlands, and it is explained mainly by climatic seasonality. These environmental variables have not been used to explain snakebite incidence, where most studies use average

precipitation and temperature as climatic incidence predictors (62,73–76). We want to emphasize the importance of understanding venomous snakes' ecology determine the environmental factors associated with snakebite.

Spatial distribution of snakebite risk in Colombia

Most of the country is under high snakebite envenoming risk (Fig 1). Higher risk values tend to cluster in departments where *Bothrops atrox* is distributed (Fig 2), where population density road infrastructure, and urbanization are low, and indigenous populations are usual (77–79). We have two hypotheses that can explain this pattern: i) Socioeconomic disparities can increase snakebite risk: Low urbanization has been demonstrated to be related with snakebite incidence (4,33,37,39,54,73,80). In addition, the intensification of anthropogenic activity (e.g., deforestation and expansion of crops lands) can also increase the risk of snakebite (37,81). ii) Biological factors can increase snakebite risk: *B. atrox* and *B. asper* are different species, thus biological variables vary among them. For example, *B. atrox* abundance is higher close to rivers in the Brazilian Amazon: Most towns in Colombian Orinoco and Amazon basin are close to water streams because it is the primary connection between towns (82–85). Additionally, population density could be higher for *B. atrox* than for *B. asper*, and based on studies on feeding ecology, *B. atrox* seems to be more active during day than *B. asper* (44,86,87). These differences can explain the higher risk in these departments, but sadly that biological data is absent in Colombia.

We believe that venomous snakes’ natural history can help to understand better snakebite (9). Studies in trophic ecology and habitat usage can help to determine risky micro-habitat conditions which can be targeted to prevent snakebite (44,86,88). In addition, reproduction studies can stablish mechanistically the temporal patterns of snakebite (62,89). Rainfall acts as a limiting resource in Colombia by decreasing incidence in marked dry seasons, which was demonstrated by mathematical models (90). Sadly, these information of venomous snakes is scarce in this country, so more robust mechanistic hypothesis behind epidemiological analyses cannot be stablished (34).

Underestimation of snakebite threat

Mortality rate in a population that do not receive antivenom can increase severely (2,5,6,91–93). Thus, if cases are underreported deaths will be critically underestimated. Treatment by antivenom is not distributed evenly, causing that places with low reporting rates have no antivenom availability (2,6,91–93). Our framework can be used to enhance antivenom availability and decrease mortality (12,42,94–96).

We estimated that 68% of the cou[illegible]y is located at more than two hours from the nearest medical center, while 36% of the country is more than 12 hours faraway from these centers (View Fig S4). Thus, there are areas where time between envenomation and medical attention is high, resulting in an increased burden and underreporting (3,92). Colombian health authorities have made a considerable effort to enhance the reporting system (37), but there is still a considerable

underestimation (View Fig 3 and Fig 4): Snakebite is a life-threatening public health problem that is still neglected, and our approach can help to quantify its threat.

Limitations

Although our proposed model can estimate snakebite' incidence using modern mathematical tools, we want to discuss a few limitations that can be improved. First, our risk estimation based on snakes' distribution is a significant contribution, but environmental niche modeling is limited by the quality of the species presence records. Several areas in Colombia and worldwide have limited accessibility, thus biological data may be scarce (View Fig S1) (97). The maximum entropy algorithm has shown good performance estimating distributions for species with incomplete presence data (50,98–100), but including abundance information may improve reliability.

Second, the assumption that the exposed population is the total rural population may not be accurate. Several municipalities have broad elevation gradients, where population is heterogeneous. Additionally, extreme urbanization would decrease the abundance of venomous snakes (101–103). Including more socio-economic variables can help to don't overlook this heterogeneity, but our model philosophy is to estimate underreporting by using basic available data and a strong and reliable statistical method, making it useful in other countries. Our statistical framework is one of the most current tools in model fitting, and it has been used successfully to asses snakebite mortality in India (104). Even so our modeling approach has several

strengths, we want to stress that it will never substitute a correct surveillance that is responsibility of public health instances.

ACKNOWLEDGMENTS

We thank to the natural history museums of the Universidad Industrial de Santander (UIS), Universidad de los Andes, Institución Universitaria ITM, Universidad del Tolima, Universidad del Valle, Universidad de la Salle (MLS), Instituto Nacional de Salud (INS), Universidad del Magdalena and Universidad Javeriana. Also, to all collections curators who gave access to specimen records, especially Martha Calderón-Espinosa and John D. Lynch (Universidad Nacional de Colombia - ICN), Andres R. Acosta-Galvis (Research institute von Alexander von Humboldt - IAvH), Fernando Sarmiento-Parra and Julieth S. Cardenas-Hincapie (MLS), Martha Patricia Ramírez and Elson Meneses-Pelayo (UIS), Juan Manuel Daza (Universidad de Antioquia - MHUA), as well as all colleagues who kindly share with us their unpublished specimen records, especially Juan José Torres (INS) and Sergio Cubides Cubillos (Butantan Institute). Finally, we thank to Minciencias and Universidad de los Andes for funding this study.

DATA SHARING

All data and code used in this study will be deposited in a recommended repository by the journal.

CONFLICT OF INTEREST

The authors declare no conflict of interest.

REFERENCES

1. Rojas G, Bogarin G, Gutierrez JM. Snakebite mortality in Costa Rica. Toxicon. 1997 Nov;35(11):1639–43.

2. Fernandez P, Gutierrez JM. Mortality due to snakebite envenomation in Costa Rica (1993-2006). Toxicon. 2008 Sep;52(3):530–3.

3. Chippaux JP. Snakebite envenomation turns again into a neglected tropical disease! J Venom Anim Toxins Incl Trop Dis. 2017;23(1):1–2.

4. Kasturiratne A, Wickremasinghe AR, de Silva N, Gunawardena NK, Pathmeswaran A, Premaratna R, et al. The Global Burden of Snakebite: A Literature Analysis and Modelling Based on Regional Estimates of Envenoming and Deaths. Winkel K, editor. PLoS Med [Internet]. 2008 Nov 4 [cited 2020 Mar 1];5(11):e218. Available from: https://dx.plos.org/10.1371/journal.pmed.0050218

5. Lalloo DG, Theakston RDG. Snake antivenoms. Vol. 41, Journal of Toxicology - Clinical Toxicology. 2003. p. 277–90.

6. Otero R, Tobón GS, Gómez LF, Osorio R, Valderrama R, Hoyos D, et al. Accidente ofídico en Antioquia y Chocó. Acta Médica Colomb. 1992;17(4):229–49.

7. Angarita-Sierra T, Montañez-Méndez A, Toro-Sánchez T, Rodríguez-Vargas A. A case of envenomation by the false fer-de-lance snake Leptodeira annulata (Linnaeus, 1758) in the department of La Guajira, Colombia.

Biomedica [Internet]. 2020 Mar 1 [cited 2021 Jun 9];40(1):20–6. Available from: https://doi.org/10.7705/biomedica.4773

8. WHO. Rabies and envenomings : a neglected public health issue : report of a Consultative Meeting. Who [Internet]. 2007;(January):32. Available from: http://www.who.int/bloodproducts/animal_sera/Rabies.pdf

9. Gutierrez JM, Williams D, Fan HW, Warrell DA. Snakebite envenoming from a global perspective: Towards an integrated approach. Toxicon. 2010 Dec;56(7):1223–35.

10. Gutiérrez JM, Warrell DA, Williams DJ, Jensen S, Brown N, Calvete JJ, et al. The Need for Full Integration of Snakebite Envenoming within a Global Strategy to Combat the Neglected Tropical Diseases: The Way Forward. PLoS Negl Trop Dis [Internet]. 2013 Jun 13;7(6):e2162. Available from: https://doi.org/10.1371/journal.pntd.0002162

11. Otero R, Fonnegra R, Jimenez SL, Nunez V, Evans N, Alzate SP, et al. Snakebites and ethnobotany in the northwest region of Colombia: Part I: traditional use of plants. J Ethnopharmacol. 2000 Aug;71(3):493–504.

12. Gutierrez JM, Fan HW, Silvera CLM, Angulo Y. Stability, distribution and use of antivenoms for snakebite envenomation in Latin America: report of a workshop. Toxicon. 2009 May;53(6):625–30.

13. Gutiérrez JM. Improving antivenom availability and accessibility: Science, technology, and beyond. Toxicon [Internet]. 2012 Sep 15 [cited 2020 Mar

1];60(4):676–87. Available from: http://www.ncbi.nlm.nih.gov/pubmed/22781134

14. World health organization. Snakebite envenoming a strategy for prevention and control [Internet]. Geneva: World health organization; 2019 [cited 2020 Nov 20]. Available from: http://apps.who.int/bookorders.

15. Hürlimann E, Schur N, Boutsika K, Stensgaard AS, de Himpsl ML, Ziegelbauer K, et al. Toward an open-access global database for mapping, control, and surveillance of neglected tropical diseases. PLoS Negl Trop Dis. 2011 Dec;5(12).

16. Bravo-Vega CA, Cordovez JM, Renjifo-Ibáñez C, Santos-Vega M, Sasa M. Estimating snakebite incidence from mathematical models: A test in Costa Rica. Bottazzi ME, editor. PLoS Negl Trop Dis [Internet]. 2019 Dec 2 [cited 2019 Dec 11];13(12):e0007914. Available from: https://dx.plos.org/10.1371/journal.pntd.0007914

17. Hossain J, Biswas A, Rahman F, Mashreky SR, Dalal K, Rahman A. Snakebite Epidemiology in Bangladesh—A National Community Based Health and Injury Survey. Health (Irvine Calif) [Internet]. 2016 Mar 15 [cited 2018 Oct 18];08(05):479–86. Available from: http://www.scirp.org/journal/doi.aspx?DOI=10.4236/health.2016.85051

18. Sinha A, Bhattacharya S, Ram R, Dasgupta U, Ram A, Majumder D. Epidemiological profile of snake bite in South 24 Parganas district of West Bengal with focus on underreporting of snake bite deaths. Indian J Public

Health [Internet]. 2014 [cited 2020 Mar 2];58(1):17. Available from: http://www.ijph.in/text.asp?2014/58/1/17/128158

19. Yañez-Arenas C, Peterson AT, Mokondoko P, Rojas-Soto O, Martínez-Meyer E. The Use of Ecological Niche Modeling to Infer Potential Risk Areas of Snakebite in the Mexican State of Veracruz. PLoS One [Internet]. 2014 Jun 25;9(6):e100957. Available from: https://doi.org/10.1371/journal.pone.0100957

20. Yañez-Arenas C, Townsend Peterson A, Rodríguez-Medina K, Barve N. Mapping current and future potential snakebite risk in the new world. Clim Change [Internet]. 2016;134(4):697–711. Available from: https://doi.org/10.1007/s10584-015-1544-6

21. Ferro C, López M, Fuya P, Lugo L, Cordovez JM, González C. Spatial Distribution of Sand Fly Vectors and Eco-Epidemiology of Cutaneous Leishmaniasis Transmission in Colombia. PLoS One [Internet]. 2015 Oct 2;10(10):e0139391. Available from: https://doi.org/10.1371/journal.pone.0139391

22. Sinka ME, Rubio-Palis Y, Manguin S, Patil AP, Temperley WH, Gething PW, et al. The dominant Anopheles vectors of human malaria in the Americas: occurrence data, distribution maps and bionomic précis. Parasit Vectors [Internet]. 2010;3(1):72. Available from: https://doi.org/10.1186/1756-3305-3-72

23. Uetz P, Hošek J. THE REPTILE DATABASE [Internet]. [cited 2018 Oct 18].

Available from: http://www.reptile-database.org/

24. Dunn ER. Los Géneros de Anfibios y reptiles de Colombia, III: Tercera Parte: Reptiles; Orden de las Serpientes. Caldasia; Vol 3, Núm 12 [Internet]. 1944 Oct 1; Available from: https://revistas.unal.edu.co/index.php/cal/article/view/32115

25. Campbell JA, Lamar WW. The venomous reptiles of the Western Hemisphere. United States of America: Comstock Pub. Associates; 2004. 870 p.

26. Rodríguez-Vargas AL, Rodriguez-Buitrago J, Diaz GJ. Comportamiento general de los accidentes provocados por animales venenosos en Colombia, 2006-2010. Rev salud pública. 2012;14(6):1001–9.

27. Patiño RO, Hernández RV, Osorio RG, Posada LE. Programa de atención primaria del accidente ofídico: una propuesta para Colombia. Iatreia. 1992;5(2):96–102.

28. Otero-Patino R. Epidemiological, clinical and therapeutic aspects of Bothrops asper bites. Toxicon. 2009 Dec;54(7):998–1011.

29. Charry-Restrepo H. Epidemiología del accidente ofídico en Colombia. Temas de Toxinologia. 2006;1–14.

30. Sasa M, Vazquez S. Snakebite envenomation in Costa Rica: a revision of incidence in the decade 1990-2000. Toxicon. 2003 Jan;41(1):19–22.

31. Valencia J, Garzón-Tello K, Barragán-Paladínes M. Serpientes venenosas

del Ecuador: sistemática, taxonomía, historia natural, conservación. Envenenamiento y aspectos antropológicos [Internet]. 1st ed. Quito: Fundación Herpetológica Gustavo Orcés; 2016 [cited 2018 Oct 18]. Available from: http://biblioteca.udla.edu.ec/client/en_US/default/search/detailnonmodal?qu=Barragán-Paladines%2C+María+Elena&d=ent%3A%2F%2FSD_ILS%2F0%2FSD_ILS%3A29363~~0&ic=true&te=ILS&ps=300

32. Sant'Ana Malaque CM, Gutiérrez JM. Snakebite Envenomation in Central and South America. In: Critical Care Toxicology. Cham: Springer International Publishing; 2015. p. 1–22.

33. Chippaux J-P. Incidence and mortality due to snakebite in the Americas. Gutiérrez JM, editor. PLoS Negl Trop Dis [Internet]. 2017 Jun 21 [cited 2018 Sep 7];11(6):e0005662. Available from: http://dx.plos.org/10.1371/journal.pntd.0005662

34. Lynch JD. El contexto de las serpientes en Colombia con un análisis de las amenazas en contra de su conservación. Rev Colomb Cienc [Internet]. 2012;36(140):435–49. Available from: http://www.scielo.org.co/pdf/racefn/v36n140/v36n140a09.pdf

35. Lynch JD, Angarita-Sierra T, Ruiz-Gómez FJ. Programa nacional para la conservación de las serpientes presentes en Colombia. Bogotá: © Ministerio de Ambiente y Desarrollo Sostenible, Colombia © Universidad Nacional de

Colombia. Instituto de Ciencias Naturales © Instituto Nacional de Salud.; 2016.

36. Nuñez León LJ. Informe Del Evento Accidente Ofidico, Colombia, 2016. Inst Nac Salud- SIVIGILA. 2016;33.

37. Nuñez León LJ, Camero-Ramos G, Gutierrez JM. Epidemiology of snakebites in Colombia (2008-2016). Rev Salud Pública [Internet]. 2020 May 30 [cited 2020 Nov 20];22(3):1–8. Available from: https://doi.org/10.15446/rsap.V22n3.87005

38. Castrillón-Estrada DF, Acosta Vélez JG, Hernández-Ruiz EA, Palacio LMA. Envenenamiento ofídico. Salud Uninorte. 2007;23(1):96–111.

39. Hansson E, Cuadra S, Oudin A, de Jong K, Stroh E, Torén K, et al. Mapping Snakebite Epidemiology in Nicaragua – Pitfalls and Possible Solutions. PLoS Negl Trop Dis [Internet]. 2010 Nov 23;4(11):e896. Available from: https://doi.org/10.1371/journal.pntd.0000896

40. Guerrero R, Gallego AI, Becerril-Montekio V, Vásquez J. Sistema de salud de Colombia . Vol. 53, Salud Pública de México . scielomx ; 2011. p. s144–55.

41. Gutiérrez JM, Fan HW. Improving the control of snakebite envenomation in Latin America and the Caribbean: A discussion on pending issues [Internet]. Vol. 112, Transactions of the Royal Society of Tropical Medicine and Hygiene. Oxford University Press; 2018 [cited 2020 Oct 1]. p. 523–6.

Available from: https://academic.oup.com/trstmh/article/112/12/523/5096868

42. Gutiérrez JM, Theakston RDG, Warrell DA. Confronting the Neglected Problem of Snake Bite Envenoming: The Need for a Global Partnership. PLOS Med [Internet]. 2006 Jun 6;3(6):e150. Available from: https://doi.org/10.1371/journal.pmed.0030150

43. de Valpine P, Turek D, Paciorek CJ, Anderson-Bergman C, Lang DT, Bodik R. Programming With Models: Writing Statistical Algorithms for General Model Structures With NIMBLE. J Comput Graph Stat [Internet]. 2017 Apr 3 [cited 2020 Nov 24];26(2):403–13. Available from: https://www.tandfonline.com/doi/abs/10.1080/10618600.2016.1172487

44. Sasa M, Wasko DK, Lamar WW. Natural history of the terciopelo Bothrops asper (Serpentes: Viperidae) in Costa Rica. Toxicon. 2009 Dec;54(7):904–22.

45. Graham CH, Ron SR, Santos JC, Schneider CJ, Moritz C. INTEGRATING PHYLOGENETICS AND ENVIRONMENTAL NICHE MODELS TO EXPLORE SPECIATION MECHANISMS IN DENDROBATID FROGS. Evolution (N Y) [Internet]. 2004 Sep 2 [cited 2018 Oct 18];58(8):1781. Available from: http://www.bioone.org/perlserv/?request=get-abstract&doi=10.1554%2F03-274

46. Hijmans RJ, Cameron SE, Parra JL, Jones PG, Jarvis A. Very high resolution interpolated climate surfaces for global land areas. Int J Climatol [Internet]. 2005 Dec 1 [cited 2018 Oct 18];25(15):1965–78. Available from:

http://doi.wiley.com/10.1002/joc.1276

47. R Development Core Team. R: A Language and Environment for Statistical Computing [Internet]. Vienna, Austria; 2008. Available from: http://www.r-project.org

48. Leroy B, Meynard CN, Bellard C, Courchamp F. virtualspecies, an R package to generate virtual species distributions. Ecography (Cop) [Internet]. 2015 May 13;39(6):599–607. Available from: https://doi.org/10.1111/ecog.01388

49. De Marco P, Nóbrega CC. Evaluating collinearity effects on species distribution models: An approach based on virtual species simulation. Bosso L, editor. PLoS One [Internet]. 2018 Sep 11 [cited 2020 Jan 23];13(9):e0202403. Available from: https://dx.plos.org/10.1371/journal.pone.0202403

50. Phillips SJ, Anderson RP, Schapire RE. Maximum entropy modeling of species geographic distributions. Ecol Modell [Internet]. 2006;190(3):231–59. Available from: http://www.sciencedirect.com/science/article/pii/S030438000500267X

51. Phillips SJ, Anderson RP, Dudík M, Schapire RE, Blair ME. Opening the black box: an open-source release of Maxent. Ecography (Cop) [Internet]. 2017 Jul 1 [cited 2020 Nov 24];40(7):887–93. Available from: http://doi.wiley.com/10.1111/ecog.03049

52. Sokal RR, Rohlf FJ. Biometry : the principles and practice of statistics in biological research. W.H. Freeman; 1995. 887 p.

53. Mohapatra B, Warrell DA, Suraweera W, Bhatia P, Dhingra N, Jotkar RM, et al. Snakebite Mortality in India: A Nationally Representative Mortality Survey. Gyapong JO, editor. PLoS Negl Trop Dis [Internet]. 2011 Apr 12 [cited 2018 Oct 18];5(4):e1018. Available from: http://www.ncbi.nlm.nih.gov/pubmed/21532748

54. Harrison RA, Hargreaves A, Wagstaff SC, Faragher B, Lalloo DG. Snake envenoming: a disease of poverty. PLoS Negl Trop Dis. 2009 Dec;3(12):e569.

55. Programme WF. Colombia Road Network (main roads) [Internet]. WFP GeoNode. 2021 [cited 2021 Jan 16]. Available from: https://geonode.wfp.org/layers/geonode:col_trs_roads_osm/metadata_detail

56. Food and Agriculture Organization of the United Nations. Land Cover of Colombia [Internet]. GeoNetwork - The portal to spatial data and information. [cited 2021 Jan 16]. Available from: http://www.fao.org/geonetwork/srv/en/metadata.show?id=37154&currTab=simple

57. Instituto Geográfico Agustin Codazzi. Major Rivers, Colombia [Internet]. [Shapefile]. 2011 [cited 2021 Jan 16]. Available from: https://earthworks.stanford.edu/catalog/tufts-colombia-major-rivers-11

58. Hijmans RJ. Geographic Data Analysis and Modeling [R package raster version 3.4-5]. R Packag [Internet]. 2020 Nov 14 [cited 2021 Jan 16]; Available from: https://cran.r-project.org/package=raster

59. PERFETTI DEL CORRAL Director M, LUZ CARDENAS FONSECA Secretaria General Directores M, Efraín Freire Delgado E, Paola Gómez Acosta A, Buitrago Hoyos G, Ricardo Valenzuela Gutiérrez R, et al. MANUAL DE USO DEL MARCO GEOESTADÍSTICO NACIONAL DANE [Internet]. 2018 [cited 2018 Sep 21]. Available from: https://www.sen.gov.co/files/RegulacionEstadistica/Manual_MGN.pdf

60. van Etten J. R package gdistance: Distances and routes on geographical grids. J Stat Softw. 2017;76(1).

61. Stoner O, Economou T, Drummond Marques da Silva G. A Hierarchical Framework for Correcting Under-Reporting in Count Data. J Am Stat Assoc [Internet]. 2019 Oct 2 [cited 2022 Jan 24];114(528):1481–92. Available from: https://www.tandfonline.com/doi/abs/10.1080/01621459.2019.1573732

62. Chaves LF, Chuang T-W, Sasa M, Gutiérrez JM. Snakebites are associated with poverty, weather fluctuations, and El Niño. Sci Adv [Internet]. 2015 Sep 1;1(8). Available from: http://advances.sciencemag.org/content/1/8/e1500249.abstract

63. Tchoffo D, Kamgno J, Kekeunou S, Yadufashije C, Nana Djeunga HC, Nkwescheu AS. High snakebite underreporting rate in the Centre Region of Cameroon: An observational study. BMC Public Health. 2019 Aug 3;19(1):1–

7.

64. Pérez Gerson. Dimensión espacial de la pobreza en Colombia. Doc Trab sobre Econ Reg [Internet]. 2005;(54):54. Available from: http://www.banrep.gov.co/sites/default/files/publicaciones/archivos/DTSER-54.pdf

65. Ponisio LC, Valpine P, Michaud N, Turek D. One size does not fit all: Customizing MCMC methods for hierarchical models using NIMBLE. Ecol Evol [Internet]. 2020 Mar 14 [cited 2020 Nov 24];10(5):2385–416. Available from: https://onlinelibrary.wiley.com/doi/abs/10.1002/ece3.6053

66. Vats D, Knudson C. Revisiting the Gelman-Rubin Diagnostic. 2018 Dec 21 [cited 2020 Nov 24]; Available from: http://arxiv.org/abs/1812.09384

67. Gelman A, Rubin DB. Inference from iterative simulation using multiple sequences. Stat Sci [Internet]. 1992 [cited 2020 Nov 24];7(4):457–72. Available from: https://projecteuclid.org/euclid.ss/1177011136

68. Kularatne AM, Silva A, Maduwage K, Ratnayake I, Walathara C, Ratnayake C, et al. Victims' response to snakebite and socio-epidemiological factors of 1018 snakebites in a tertiary care hospital in Sri Lanka. Wilderness Environ Med. 2014 Mar;25(1):35–40.

69. Ramírez JC, De Aguas JM. Escalafón de la competitividad de los departamentos de Colombia 2015: Versión en edición. Of la CEPAL en Colomb. 2015;107.

70. Abatzoglou JT, Dobrowski SZ, Parks SA, Hegewisch KC. TerraClimate, a high-resolution global dataset of monthly climate and climatic water balance from 1958-2015. Sci Data. 2018 Jan 9;5(1):1–12.

71. Mischler P, Kearney M, McCarroll JC, Scholte RGC, Vounatsou P, Malone JB. Environmental and socio-economic risk modelling for Chagas disease in Bolivia. Geospat Health. 2012 Sep;6(3):S59-66.

72. Peterson AT, Papeş M, Soberón J. Rethinking receiver operating characteristic analysis applications in ecological niche modeling. Ecol Modell [Internet]. 2008;213(1):63–72. Available from: http://www.sciencedirect.com/science/article/pii/S0304380007006163

73. Hansson E, Sasa M, Mattisson K, Robles A, Gutiérrez JM. Using Geographical Information Systems to Identify Populations in Need of Improved Accessibility to Antivenom Treatment for Snakebite Envenoming in Costa Rica. PLoS Negl Trop Dis [Internet]. 2013 Jan 31;7(1):e2009. Available from: https://doi.org/10.1371/journal.pntd.0002009

74. Ediriweera DS, Diggle PJ, Kasturiratne A, Pathmeswaran A, Gunawardena NK, Jayamanne SF, et al. Evaluating temporal patterns of snakebite in Sri Lanka: the potential for higher snakebite burdens with climate change. Int J Epidemiol [Internet]. 2018 Sep 11 [cited 2018 Oct 7]; Available from: https://academic.oup.com/ije/advance-article/doi/10.1093/ije/dyy188/5094971

75. Molesworth AM, Harrison R, David R, Theakston G, Lalloo DG. Geographic

information system mapping of snakebite incidence in northern Ghana and Nigeria using environmental indicators: a preliminary study. Trans R Soc Trop Med Hyg [Internet]. 2003 Mar [cited 2018 Oct 7];97(2):188–92. Available from: https://academic.oup.com/trstmh/article-lookup/doi/10.1016/S0035-9203(03)90115-5

76. Shashar S, Yitshak-Sade M, Sonkin R, Novack V, Jaffe E. The Association Between Heat Waves and Other Meteorological Parameters and Snakebites: Israel National Study. J Emerg Med [Internet]. 2018 Jun 1;54(6):819–26. Available from: https://www.sciencedirect.com/science/article/pii/S0736467918301112

77. Pérez GJ. La infraestructura del transporte vial y la movilización de carga en Colombia [Internet]. Documentos de trabajo sobre economia regional. Cartagena: Banco de la república; 2005 [cited 2019 Dec 11]. Available from: http://www.banrep.gov.co/docum/Lectura_finanzas/pdf/DTSER-64.pdf

78. Grupo de Planeación en la Salud Pública - Ministerio de salud. CONTEXTO GENERAL DE LOS PUEBLOS INDÍGENAS: ASPECTOS SOCIO CULTURALES, DEMOGRÁFICOS, AMBIENTALES, TERRITORIALES Y DE SALUD. Bogotá, D.C: Ministerio de salud; 2017.

79. Dirección de Desarrollo Rural Sostenible - DDRS. Departamento Nacional de Planeación. MISIÓN PARA LA TRANSFORMACIÓN DEL CAMPO Definición de Categorías de Ruralidad. 2014.

80. da Silva CJ, Jorge MT, Ribeiro LA. Epidemiology of snakebite in a central

region of Brazil. Toxicon. 2003 Feb;41(2):251–5.

81. Fry BG. Snakebite: When the human touch becomes a bad touch [Internet]. Vol. 10, Toxins. MDPI AG; 2018 [cited 2021 Feb 1]. p. 170. Available from: /pmc/articles/PMC5923336/?report=abstract

82. Alcântara JA, Bernarde PS, Sachett J, da Silva AM, Valente SF, Peixoto HM, et al. Stepping into a dangerous quagmire: Macroecological determinants of Bothrops envenomings, Brazilian Amazon. Gutiérrez JM, editor. PLoS One [Internet]. 2018 Dec 6 [cited 2019 Dec 11];13(12):e0208532. Available from: http://dx.plos.org/10.1371/journal.pone.0208532

83. Fraga R de, Magnusson WE, Abrahão CR, Sanaiotti T, Lima AP. Habitat Selection by Bothrops atrox (Serpentes: Viperidae) in Central Amazonia, Brazil. Copeia. 2013 Dec 30;2013(4):684–90.

84. Meisel A, Leonardo R, Andrés B, Jabba S. Geografía económica de la Amazonia Colombiana. Documentos de trabajo sobre economía regional; 2013.

85. Armenteras D, Rudas G, Rodriguez N, Sua S, Romero M. Patterns and causes of deforestation in the Colombian Amazon. Ecol Indic. 2006 Apr;6(2):353–68.

86. M Oliveira E, Martins M. When and where to find a pitviper: activity patterns and habitat use of the lancehead, Bothrops atrox, in Central Amazonia, Brazil. Vol. 8, Herpetological Natural History. 2001. 101–109 p.

87. Wasko DK, Sasa M. Habitat Selection of the Terciopelo (Serpentes: Viperidae: Bothrops asper) in a Lowland Rainforest in Costa Rica. https://doi.org/101655/08-064R21 [Internet]. 2010 Jun 1 [cited 2021 Oct 21];66(2):148–58. Available from: https://bioone.org/journals/herpetologica/volume-66/issue-2/08-064R2.1/Habitat-Selection-of-the-Terciopelo-Serpentes--Viperidae--Bothrops/10.1655/08-064R2.1.full

88. Wasko DK, Sasa M. Food resources influence spatial ecology, habitat selection, and foraging behavior in an ambush-hunting snake (Viperidae: Bothrops asper): an experimental study. Zoology [Internet]. 2012 Jun [cited 2018 Oct 18];115(3):179–87. Available from: http://www.ncbi.nlm.nih.gov/pubmed/22440190

89. Solórzano A, Cerdas L. Reproductive Biology and Distribution of the Terciopelo, Bothrops asper Garman (Serpentes: Viperidae), in Costa Rica. Herpetologica [Internet]. 1989;45(4):444–50. Available from: http://www.jstor.org/stable/3892835

90. Bravo-Vega CI, Santos-Vega MI, Manuel Cordovez JI. Disentangling snakebite dynamics in Colombia: How does rainfall and temperature drive snakebite temporal patterns? Ainsworth SR, editor. PLoS Negl Trop Dis [Internet]. 2022 Mar 31 [cited 2022 Apr 5];16(3):e0010270. Available from: https://journals.plos.org/plosntds/article?id=10.1371/journal.pntd.0010270

91. Gerardo CJ, Evans CS, Kuchibhatla M, Mando-Vandrick J, Drake WG, Yen

M, et al. Time to antivenom administration is not associated with total antivenom dose administered in a copperhead-predominant snakebite population. Acad Emerg Med. 2015 Mar;22(3):308–14.

92. Gerardo CJ, Evans CS, Kuchibhatla M, Drake WG, Mando-Vandrick JD, Yen M, et al. Time to Antivenom Administration in Snakebite. Ann Emerg Med [Internet]. 2013 Oct 1 [cited 2018 Sep 19];62(4):S44. Available from: https://linkinghub.elsevier.com/retrieve/pii/S0196064413010822

93. LÓPEZ NILSON, LOPERA CLARA RÁ. Características de los pacientes con accidente ofídico y complicaciones infecciosas atendidos en el Hospital Pablo Tobón Uribe entre los años 2000 y 2006. Acta Médica Colomb. 2008;33:127–30.

94. Sarmiento K. Aspectos biomédicos del accidente ofídico. Univ Méd Bogotá Colomb. 2012;53 (1):68–85.

95. Pineda D, Ghotme K, Aldeco ME, Montoya P. Accidentes ofídicos en Yopal y Leticia, Colombia, 1996-1997. Biomédica [Internet]. 2002;22(1):14. Available from: http://www.revistabiomedica.org/index.php/biomedica/article/view/1135

96. Habib AG, Kuznik A, Hamza M, Abdullahi MI, Chedi BA, Chippaux J-P, et al. Snakebite is Under Appreciated: Appraisal of Burden from West Africa. PLoS Negl Trop Dis [Internet]. 2015;9(9):1–8. Available from: https://doi.org/10.1371/journal.pntd.0004088

97. Andrade-C. MG. ESTADO DEL CONOCIMIENTO DE LA BIODIVERSIDAD

EN COLOMBIA Y SUS AMENAZAS. CONSIDERACIONES PARA FORTALECER LA INTERACCIÓN CIENCIA-POLÍTICA . Vol. 35, Revista de la Academia Colombiana de Ciencias Exactas, Físicas y Naturales . scieloco ; 2011. p. 491–507.

98. Phillips SJ, Dudík M. Modeling of species distributions with Maxent: new extensions and a comprehensive evaluation. Ecography (Cop) [Internet]. 2008 Apr 1 [cited 2018 Sep 20];31(2):161–75. Available from: http://doi.wiley.com/10.1111/j.0906-7590.2008.5203.x

99. Kumar S, Neven LG, Zhu H, Zhang R. Assessing the Global Risk of Establishment of Cydia pomonella (Lepidoptera: Tortricidae) using CLIMEX and MaxEnt Niche Models. J Econ Entomol [Internet]. 2015 Aug [cited 2018 Oct 7];108(4):1708–19. Available from: https://academic.oup.com/jee/article-lookup/doi/10.1093/jee/tov166

100. Saupe EE, Papes M, Selden PA, Vetter RS. Tracking a Medically Important Spider: Climate Change, Ecological Niche Modeling, and the Brown Recluse (Loxosceles reclusa). Moreau C, editor. PLoS One [Internet]. 2011 Mar 25 [cited 2018 Oct 7];6(3):e17731. Available from: http://dx.plos.org/10.1371/journal.pone.0017731

101. Chippaux JP. Snake-bites: appraisal of the global situation. Bull World Health Organ [Internet]. 1998 [cited 2018 Oct 7];76(5):515–24. Available from: http://www.ncbi.nlm.nih.gov/pubmed/9868843

102. França RC de, Germano CE de S, França FGR. Composição de uma

taxocenose de serpentes em uma área urbana na Mata Atlântica da Paraíba, Nordeste do Brasil. Biota Neotrop [Internet]. 2012 Sep [cited 2018 Oct 7];12(3):183–95. Available from: http://www.scielo.br/scielo.php?script=sci_arttext&pid=S1676-06032012000300019&lng=pt&tlng=pt

103. Johnson SA, Mcgarrity ME. Dealing with Snakes in Florida’s Residential Areas-Identifying Commonly Encountered Snakes 1 [Internet]. 2007 [cited 2018 Oct 7]. Available from: http://edis.ifas.

104. Suraweera W, Warrell D, Whitaker R, Menon G, Rodrigues R, Fu SH, et al. Trends in snakebite deaths in India from 2000 to 2019 in a nationally representative mortality study. Elife. 2020 Jul 1;9:1–37.

Click here to access/download
Supporting Information - Compressed/ZIP File Archive
SI.zip
