# Supplementary material for: A generalized framework for estimating snakebite underreporting using statistical models: A study in Colombia
Source: PLoS Negl Trop Dis. 2023 Feb 6;17(2):e0011117. doi: 10.1371/journal.pntd.0011117 (PMC9934346; doi:10.1371/journal.pntd.0011117)
Supplement: Attachment — Submitted filename: Response to reviewers.docx [file pntd.0011117.s010.docx]

**Reviewer #1:**

- The manuscript is well written, the theoretical foundation of the model well supported, and I consider that the work deserves to be published. However, a series of observations/suggestions included in the attached pdf file as messages could help to clarify some of the points presented by the authors.

Thank you very much for your comments. We have addressed all your suggestions in the PDF file. The quality of the manuscript has greatly improved.

- Introduction. This section needs a couple of lines clarifying the factors that can generate underreporting and which of them can be assumed in Colombia. In addition, a generalized mixed effect model is mentioned, with poverty as one of the considered factors. However, there is no previous mention of why this factor should be considered when estimating the incidence of snakebite, nor its relationship with this health problem. I suggest clarifying it in this section.

Thank you for pointing this out. We now note both in the Introduction (L 73-74) and in the Methods section (L245-249) the importance of poverty as a factor that influences snakebite underreporting. Poverty also influences snakebite incidence, but we used this variable only as an estimator of underreporting. After including your suggestion, it is now made much clearer why we decided to use poverty to estimate underreporting, and this has made the presentation of our methodology clearer and more robust. We very much appreciate this comment.

- The analysis presented match the proposed plan and the results are clear. 
  However, implementing the proposed model results in higher estimates of snakebite incidence, 10% of the number recorded in SIVIGILA. However, we are not told which or how many municipalities show this increase. Furthermore, it is unclear if there is a threshold between the model estimates and the recorded incidence data that denotes no underreporting. Or are both estimates compared by some paired test? The authors must explain this point to clarify that the model can effectively identify underreporting cases and discriminate them from cases where there is none.

Thank you for your comment. We conducted a test for all municipalities to determine whether the mean of underreporting significantly differed from zero, and we found that the mean differed from zero for all municipalities (p-values < 0.001). Thus, we concluded that our model effectively estimated underreporting for every municipality. We clarified this point in the text. Additionally, the number of yearly underreported cases was close to zero for some municipalities, and it was assumed that this minimum underreporting occurs in these municipalities. We decided to leave raw results in the text because our algorithm is new, and we were not able to find any other tool that would permit us to differentiate between municipalities with and without underreporting. We included a sentence explaining this (L329-L331)

- In Figure 3, the differences between maps 3a and 3b are not notable, possibly due to an effect of the scales of the categories used. This obscures the contrast the authors make in the text about the incidence records and their model estimates, further noted in Figure 4. The authors should consider this point to make their point clearer. In addition, since the Department of Antioquia is mentioned in the text, I suggest they indicate it on the map in Fig 3.

Thank you for pointing this out. This was also a point brought up by the other reviewer. We have modified Figure 3. We put the number of reported SIVIGILA cases, as well as a map with our estimated number of cases that are not reported. Here, the differences are notable, and this revised figure is much easier to understand than the previous figure. In addition, we indicated the location of Antioquia. This has greatly enhanced the clarity of our manuscript. We appreciate this comment.

- Conclusions are supported by the data presented. The discussion of the model and its results are pertinent. However, one aspect that is omitted is how to evaluate the results of these estimates empirically. I believe that this section would benefit from advancing how to determine that these underreporting estimates correspond to cases that effectively escape the system. Perhaps the authors have empirical data that allow such an evaluation; failing that, they could leave a possible future study to assess it.

Thank you for this suggestion. We included a new part in the discussion (L415-420) in which we call for more in-field epidemiological studies of snakebite, as well as research to explore alternatives for measuring snakebite underreporting. We have data on one municipality in Colombia, with a rate of underreporting similar to that estimated by our model; however, these data remain unpublished, and we have thus decided to refrain from presenting this underreporting estimation without it having gone through peer review. We also think that it would not be very robust to validate our algorithm with an estimate from a single municipality (i.e., N = 1). We plan on publishing the paper containing this estimate soon to strengthen our algorithm.

- As stated above, minor modifications on the manuscript will enhance clarity.  Some other comments in attached file. I recommend "Minor revision"

We appreciate these suggestions. The article has become more concise and the algorithm more robust thanks to this peer review. Thank you for reading and commenting on our work. We look forward to your feedback on the next round of revisions.

**Reviewer #2:**

- It would be necessary to ease the understanding explaining what 0.3° or 0.7° mean. Please mention what their equivalents in metric units are. Additionally, it would be necessary to explain how these grid sizes were selected. A reference used for applying the same algorithm (Yañez-Arenas et al., 2014) for a large area from North America to Colombia they used a grid of 0.05°. If this method implies the random removal of all but one observation, why to use such much larger areas to determine SNMS? Why did the authors choose to remove the large majority of their observations? This should be evaluated and improved, or valid limitations clearly explained.

Thank you for your suggestion. We analyzed different grid sizes between our proposed sizes (0.3° and 0.7°) to the proposed grid size of 0.05°, which is a pixel size of approximately 5 km × 5 km. We found that the proposed grid by Yañez-Arenas et al. (2014) greatly enhanced our maximum entropy models. We used such large-sized grids because we were afraid of using autocorrelated presence records, but the use of a smaller grid size allowed more presence records to be retained, which improved the distribution models and our SNMS estimation, including the capacity to capture information on the snakebite reported risk distribution.

We altered the grid sizes in our manuscript (L164-165) and included the Yañez-Arenas reference that used that grid size. We updated the number of records after data depuration (L171-172). We also removed Fig. S1 because the fine resolution of the grid (5 km × 5 km) at the country scale does not provide clear information on this figure.

- Relevance of SNMS results for the final under-reporting model:
  L255: It is not clear, why if the SNMS is modelling the suitable areas for the two snake species, the “pristine forests” of the amazon region would not be suitable for B. atrox? The goal of Maxent is precisely to predict the expected the distribution of the species based on some observations. Please elaborate on this issue, since that would clearly affect the estimated incidence. In relation to this, the AUC of 0.65 reported for the SNMS of B. atrox shows a very low discrimination power. This value is closer to represent randomness (0.5) than perfect predictability (1.0), thus the validity of SNMS as explanatory variable of the incidence would need to be evaluated, or a better set of variables chosen to produce that factor.

Thank you for noticing this problem. After implementing these suggested changes to our methodology, the AUC and CBI values increased for both species. The AUC value for *B. atrox* is now 0.77. This thus increased the discrimination capacity of our models. In addition, the CBI values for the distributions of both snakes were greater than 0.9, which means that they clearly deviated from a random model when only presence records were used. We greatly appreciate this suggestion, as it has greatly improved our model.

We removed the previous analysis based on regions of the country and omitted general regions, such as the “pristine forests.” We showed how both distributions vary in their spatial heterogeneity (L278-281). Suitability for *B. asper* is more heterogeneous than that for *B. atrox*, and that of the latter is rather homogeneous. This enhances the robustness of our results compared with the visual regions that were used before, many thanks for this suggestion.

- Validity of the accessibility score:
  The authors mention that there was no information on which health facilities were or not able to treat snakebite envenoming. Does it mean that all health facilities of all sizes were considered as able to treat envenoming? This should be clarified as this might result in an over estimation of the treating capacity with additional consequences for under-reporting. Additionally, for the accessibility score, it should be specified what travel speeds were used between different landcover, roads, and rivers, as well as some indication of how those speeds were chosen. All this suggests that the accessibility score might be unrealistic and would lose its representativeness and value.

Thank you for pointing this out. An expert in the public health management of snakebite (the coauthor Leonardo Nuñez Leon) selected three specific types of health facilities from the map that we used, including clinics, hospitals, and health service providers. This selection was based on his knowledge of the facilities with the capacity to maintain a supply of and administer anti-venom. We included a specific sentence to clarify this (L220-223). We also included information on the travel speeds used in each one of the maps analyzed in our accessibility information in Table S2 (L217-218). These changes have greatly clarified the methodology, many thanks.

Language:

- Please improve the English throughout the text either by having the manuscript reviewed by a native English speaker or by using an AI editor such as Instatext.

Thank you for recommending a revision of the English language. The entire manuscript has been revised by a native English-speaking editor.

- Unless you are providing information about which you are completely certain, please avoid using deterministic language that presumes the concepts to be true or definitive.

Thank you for pointing this out. We have removed all such language in the revised version of the manuscript.

Referencing:

- The use of supporting references throughout the paper seems to be unnecessarily bulked-up. There are rather simple ideas with six or more references. This is partially a problem because 50% of the references are 10 or many more years old, and some ideas are supported with what seems counterargument, e.g. L77 where two of the references are actually cross-sectional studies, and the third is a similar study in Costa Rica, where this same idea is mentioned without references.

Thank you for noticing this inconsistency and the unnecessary references. We have revised the references of the entire manuscript, and we have only used recent references. The number of references has been reduced from 104 to 87. The references have also been made more consistent.

- L384: Please beware of differences between concepts and opinions/results, and how the former should be referenced, while the latter not.

Thank you for this suggestion. We have removed the references in this line (L421), and we have revised the discussion so that concepts are supported by references whereas opinions are not supported by references.

Basic concepts:

- In L77-88 is disused the snake diversity leading finally to the selection of two species as main responsible for envenomations. The most recent reference (Nr 37) mentions 64.5% as the proportion of bites by Bothrops. This fundamental concept could be better documented including e.g. a range of percentages, especially considering how old some references are.

Thank you for pointing this out. The most recent reference (Nuñez-Leon, 2020) mentioned that value. Older studies (e.g., Otero, 2018) have suggested a value between 50 and 80% based on clinical studies. This inconsistency stems from the fact that SIVIGILA data reports that around 26% of bites are caused by unidentified snakes (Epidemiological report from SIVIGILA for 2019, which is the most robust recent report because COVID-19 affected data for 2020 and 2021). Thus, we included the range proposed by Otero (2018) (L93-94).

- It would also be necessary to clarify whether and how dry bites are considered, since they do not generate envenoming and therefore might influence incidence values if they are recorded or under-reporting if they are not.

Thank you for pointing this out. We now discuss the consideration of dry bites in the second paragraph of the section “*Snake niche modelling score map computation”* (L192-194)*.* Specifically, we note that the data indicate snakebites in which medical attention was sought; however, whether the bites ended up being dry was not specified. We also note in the section “*Model description*” that our model does not differentiate between dry bites and envenomings (L236-238).

- The threshold of 0.9 used to exclude correlated environmental layers is too high. Pearson’s correlations above 0.7 are considered already strong. This should be explained as well as the possible consequences of including highly correlated variables. Following the same example of referenced base articles, Yañez-Arenas et al. 2014, used 0.7 as threshold to remove covariates.

Thank you for this suggestion. We compared the results using the 0.9 and 0.7 thresholds and found that using a threshold of 0.7 yields better results and a simpler model. We thus used the 0.7 threshold in the manuscript per your suggestion (L 177). This has been updated in Table S1.

- L170: The description of habitat suitability modelling in text and in ‘S1 text’ is rather vague. The rationale behind the different methodological choices is lacking, especially considering the suggestion of the authors of replication in other countries. Examples of the intermediate results and the basic code to replicate those results could be included in the supplementary material files.

Thank you for this comment. We included a more detailed description and explained the reasoning behind our methodological choices thoroughly. We also have uploaded the code referred to by the reviewer to figshare so that they can be replicated by others. The link to this code is included in the article (L470-471) as well as in S1 text.

- L180: It is not clear how the reported risk is computed, and as a consequence the difference between reported incidence and reported risk. As presented in the text, reported risk would be an average incidence of several years. If a standardization with the population is used, it should be explained, since incidence is usually already standardized per 100’000 population.

Thanks for pointing this out. We conducted our calculation of reported risk by normalizing the averaged SIVIGILA-reported incidence of the rural population. We also emphasize that reported risk is in units of cases per 100.000 population per year (L188-200). This addition has helped clarify this; thank you for the comment.

- L183: If the goal is to produce a methodology that can be reproduced, it is necessary to clarify much better the comparison and selection processes done to the SNMS. Please give more detail or ideally create an SOP. Additionally, it is not clear the transit from SNMS to SNMS*.

Thank you for this suggestion. We have revised the text so that the methodology can be more easily reproduced by others; we have also made the procedure for the transition from SNMS* to SNMS clearer by updating the names of the variables (L180-206). Additionally, we included Fig. S1, which is a SOP that describes the procedure for this selection process. These revisions will make our procedure easier to reproduce by others. We have also uploaded all the code to figshare to make our code accessible.

- L206+: The term ‘real incidence’ is misleading considering that this value is an estimation based on proxy values.

Thank you for your suggestion. We changed the term from “real incidence” to “total incidence estimation”, which emphasizes that this is still an estimation based on proxy values.

- L219: The poverty index included is clearly an important factor in the computation of incidence, but it is not clear what elements were analysed as part of that index and the references do not explain it, but instead discuss the importance of poverty to snakebite. It would be preferable to keep one or two references indicating the importance of poverty to snakebite, and also a source for the index itself, as well as clarification of the parameters included in it.

Thank you for this suggestion. We have provided better explanations of the parameters that were included, including the unsatisfied basic needs (L246-248). We also updated references and reordered them so that they fit better with references 5 and 28 on the use of the poverty index on the reporting fraction of snakebite. References 50, 51 provide information on our chosen poverty index.

- In L258 & 263, is not clear what type of relationship is being suggested, if at all,  between areas with low/high number of observations and the resulting SNMS, since both concepts are mentioned simultaneously, and present in high/high, low/high, high/low and low/low combinations in the maps. If the relationship is high/high and low/low, the connection later presented between the SIVIGILA reported risk (L277: SBE/100’000 people/year, which is usually understood as incidence) and the SNMS values would be expected. Overall, these relationships should be explained more clearly.

Thank you for pointing out this problem. We agree that our definitions of incidence and risk were not robust. We have thus revised them. We now define incidence as the new cases that occur in a specific timespan (Cases/year), and the Person-time incidence rate (Cases / 100.000 population * year) is what we defined as snakebite risk. We also clarified the relationships on which you commented. We found that departments with *B. atrox* (Orinoco and Amazonian basin) form a cluster with high person-time incidence rates and high values of snake suitability (SNMS). By contrast, departments with *B. asper* show no clustering with respect to incidence and risk values. This is shown in Fig 2. In this figure, red points (departments with *B. atrox*) are clustered in the upper right area of the plot (high incidence rate and SNMS), whereas green points (*B. asper*) are distributed throughout the plot. We added additional text to clarify this point (L296-299, L301 – 306), and we expect that this should resolve any outstanding uncertainty.

- L326: Please clarify. In this analysis, was a less robust estimation done in all the places where there was no data? Or, hypothetically no analysis could be done if there were no data at all? The latter would be unnecessary to mention, since no model generates estimates without any data at all.

Thank you for your suggestion. We have revised this part of the discussion and state explicitly that our work did not estimate underreporting for departments without data (L346-349). We have also emphasized that proxy data are required for our framework to be used.

- L237: For actual replicability, it would be necessary to explain the decisions taken. For instance, why did you choose to use 4.7 million iterations? Is that number justified somehow?

Thank you for this suggestion. We have modified this part of the manuscript because these iterations cannot be justified with a reference. In each iteration, a search for the “optimum” value of the parameters was performed that maximized the likelihood of the model and data. Thus, the number of iterations varies depending on the data. The number of iterations is important for achieving convergence of the algorithm. We have revised this part of the manuscript and explained how we were able to achieve convergence after these iterations so that other researchers can replicate the framework while ensuring that convergence is achieved, as the number of iterations cannot be preset (L267-269).

- L340: The authors address the validity of the risk estimation by mentioning that AUC and BIC values were higher than 0.5. For AUC that statements does not mean much, other than the ‘direction’ of the prediction. For BIC (if it means Bayesian Information Criterion), a value of 0.5 does not represent anything by itself. Please present your results in a more rigorous and precise manner.

Thank you for pointing this out. We initially used CBI (Continuous Boyce Index), which is a statistic used for distribution models that was designed for models that work only with presence data, such as Maxent. This index ranges from -1 and 1 and provides a more robust indication of the validity of the model prediction with presence locations than AUC. Negative values of the CBI denote inconsistencies between the prediction and presence locations, and positive values close to one indicate a robust model. Thanks to your previous suggestions, we increased the AUC and CBI for both species, and the values are now closer to one. We have revised this paragraph, including the definition of both statistics and how to interpret the values (L361-370).

- L357-359: the concepts discussed are confusing and imprecise. It has been shown that ‘socio-economic disparities’ e.g. poverty are linked to higher risk of snakebite, and as referenced higher urbanization is linked to lower snakebite incidence and rurality to higher incidence, but low urbanization and intensification of anthropogenic activities are not necessarily linked to socio-economic inequality, as claimed. There are highly urbanized areas where socio-economic inequality is high as well.

Thank you for pointing this out. We were not referring to socioeconomic disparities themselves but rather to socioeconomic variables that differ among areas where *B. atrox* can be found, such as anthropogenic activity and low urbanization. Thus, we used socioeconomic variables that more accurately capture what we had originally intended (L383-389).

Acronyms:

- The acronym SNMS should be better explained. In L135 could be understood that the first S refers to ecological, which of course does not make sense, only in L255 it is clear that it means ‘Snake’.

Thank you very much for pointing this out. We agree that this could have been made clearer. We removed the concept of the snakebite envenoming risk score; we now only refer to SNMS as the snakebite niche modeling score. To enhance the consistency within the manuscript, we have provided a better explanation of the process we used to validate this score.

- In the results, even if they might be well known, please define the acronyms AUC, CBI, AICc, and BIC. Additionally, in L341 it is mentioned once the use of BIC together with AUC. Is CBI or BIC a misspelling of the other?

Thank you for this suggestion. We have defined the acronyms, and we have removed BIC because it was in fact a misspelling. We only used AUC, AICc, and CBI (Continuous Boyce Index) to evaluate our niche modeling maps.

Equations and formulas:

- The formulas are mislabelled, starting with 3.

Thank you for noticing this; we corrected the labels of the equations.

- It would be necessary to clarify how α is defined. This is particularly important since the contact rate between different human and snake populations might differ depending on any number of circumstances.

Thank you for noticing this. We have provided a better explanation of the changes made to equation 1 in reference 29 in our model, as well as how α stems from merging parameters ϴ and β of that equation. We also explained that this parameter is constant (L150-152).

- The paper used to reference the law of mass action, also includes the probability of an encounter resulting in a bite. Why was this omitted in this manuscript?

Thank you for this suggestion. We now explain why we omitted this probability.

Plots and figures:

- The 3D plot presented in Fig. S3 shows a very strong influence of accessibility on underreporting, but in comparison, a rather weak effect of poverty. However, that second relationship it is difficult to visualize. It would help to understand the results if numeric results of the models were presented (e.g. parameters and their credible intervals).

Thank you for this suggestion. We now include a table with 95% credible intervals.

- The legend describing the colour code of Fig. 4 talks about the percentage of under reporting, which is mentioned in the text to go from 5.7 to 38.7%, but here indicates 0.057 to 0.366. If this means to show percentages, it should be consistent with the text. Otherwise, it could be misunderstood as 0.057%. Additionally, if as understood, both ranges are the same, the upper limit should be equal in the text and the figure.

Thank you for pointing out these inconsistencies. We have updated the legend of Figure 4, which shows that these are in fact percentages. We also corrected the limits.

- The differences presented in paired maps in figure 3 are minimal and difficult to determine. A complementary approach could be the addition of a map presenting the difference between reported and estimated.

Thank you for noticing this. We have revised Figure 3, which now shows the reported SIVIGILA cases and the difference between our estimation and reported cases (i.e., cases that were not reported to SIVIGILA). We also revised the caption for this figure.

- It is the role of the journal, but I would like to point out how odd is to find results and comments in the legends of figures 1-3, which extend them significantly. I would prefer if the legend were used to describe the figure construction and characteristics.

Thank you for this suggestion. Other manuscripts published in PLOS NTDS have incorporated these types of legends. Nevertheless, we decided to reduce the amount of information presented in each legend and now only include a description of the figure per this suggestion.

- The article describes an interesting approach to estimate computationally snakebite under-reporting. However, the study present important weaknesses that should be addressed.

Thank you for your comments. We have addressed all your comments in the revised version of the manuscript. These revisions have greatly strengthened our paper. We really appreciate your revision of our article.
