# Supplementary material for: A generalized framework for estimating snakebite underreporting using statistical models: A study in Colombia
Source: PLoS Negl Trop Dis. 2023 Feb 6;17(2):e0011117. doi: 10.1371/journal.pntd.0011117 (PMC9934346; doi:10.1371/journal.pntd.0011117)
Supplement: Attachment — Submitted filename: Response to reviewers.docx [file pntd.0011117.s011.docx]

**Reviewer #1:** In this manuscript, the authors present relevant information for understanding snake envenoming, one of the neglected diseases with the most significant impact in the tropics. The information presented is innovative and relevant as a procedure for the statistical analysis of snakebite incidence and estimating underreporting cases is described. The autors apply this approach to Colombia as a case study. This new version has addressed many of this reviewer's initial observations and is more straightforward for PNTD readers. I only have a few minor form suggestions that I outline below. Other than that, I consider the manuscript ready for publication.

Thank you for noticing our effort including all your reviews. After including your comments, the article quality improved greatly, and we appreciate it a lot. It is nice to have reviewers that perform constructive comments as you have done, and thanks to including and working on these comments more robust science can be achieved. We appreciate it a lot, and acknowledge your time reading our manuscript and helping us improving its quality.

L121: “Our paper describes an algorithm for estimating underreporting using Colombia as a case study”. Add a line or paragraph before to introduce the problem of underreporting of the snakebite in Colombia

Thank you very much for your suggestion. We included two sentences (L 117-120) in previous paragraph to introduce underreporting concept in Colombia and also to justify why is it expected to exist. Now the justification of our research objective is clearer, we appreciate it a lot.

122-131 These lines are somewhat repetitive of those explained in the methods section.  I suggest simply stating that para proposes a statistical framework to estimate snakebite.  Consider:
“Our article describes an algorithm to estimate underreporting using Colombia as a case study. For this, we developed a model that considers the risk of snakebites based on the distributions of the most important species from the epidemiological point of view. We tested its performance using the incidence registered in the country's public health system. Using a generalized model incorporating this risk estimator and other factors associated with snakebite incidence, we estimated the reported fraction of total cases and the underreporting of cases on spatial and time scales”.

Thank you very much for your suggestion. We changed the paragraph that finalizes introduction, and we simplified it. We used your suggestion and changed two parts: First, we said that the generalized model uses the risk estimators and other factors associated with snakebite underreporting, instead of factors associated with snakebite incidence. Finally, we changed the last sentence to “we estimated the spatial distribution of snakebite cases underreporting”. This is because our underreporting estimation is not on timescale, but only on spatial scale. If we had yearly data of poverty, and the yearly changes of roads and land coverage, we could also perform a time-scale estimation. We feel that with this introduction closure, the objective of the research is more evident, and it is not repetitive with methods. We appreciate your suggestion a lot (L124 – 130).

311     " the multivariate index was 1 (53,54), indicating convergence" .       Since the interpretation of the index is described in those references, mark them at the end of the sentence.  Ej:  the multivariate index was 1, indicating convergence (53,54)

Thank you for your comment. We implemented your suggestion (L 310).

327-330 Consider delineating the regions with the greatest change in Figure 3B

Thanks you for your comment. We considered and delineated the regions with the greatest change in Figure 3B. We also included Choco as another region. Now reader can look easier to the changes we are pointing at.

363-365 " Given that these data are available for several countries, snakebite burden can be approximated quickly with our approach."    Consider: Since these data are available for several countries, the snakebite burden can be rapidly approximated with our approach.

Thank you very much for your suggestion. We included your proposal (L362-364).

396-405  The authors suggest possible differences in density between B. asper and B. atrox, attributing it to differences in their biology. However, differences in density can also result from the relationship with humans: higher mortality rates could be expected in more human-populated environments, that in Colombia roughly coincide with B. asper distribution.

Thank you for your point. We readed that paragraph and decided that our hypotheses that explain the differences of snakebite risk between both places’ benefits from your point. Thus, we included explicitly that ecological differences may cause higher abundance of *B. atrox*, and that *B. asper* inhabits areas with higher human population density; thus, its mortality will increase, and population density will became lower than that of *B. atrox* (L 401-407)

428-430 " We estimated that 68% of the country is located more than two hours away from the nearest medical center, and 36% of the country is more than 12 hours away from medical centers".   But how much does this represent in terms of human population?

Thank you for noticing this lack of information. We assumed a homogeneous rural human population in each municipality and determined the areas above two and 12 hours. Then, we estimated the rural population in each area. We included this information in the paragraph, and now it became more clearer the real effect of this long distance over snakebite risk (L432-436)
